# First genome-wide association study investigating blood pressure and renal traits in domestic cats

**DOI:** 10.1038/s41598-022-05494-3

**Published:** 2022-02-03

**Authors:** R. E. Jepson, H. Warren, M. D. Wallace, H. M. Syme, J. Elliott, P. B. Munroe

**Affiliations:** 1 Department of Clinical Services and Sciences, Royal Veterinary College, London, UK; 2 Department of Comparative Biomedical Sciences, Royal Veterinary College, London, UK; 3 Department of Clinical Pharmacology, Barts and the London School of Medicine and Dentistry, Queen Mary University of London, London, UK; 4 Nuffield Department of Medicine, Wellcome Centre for Human Genetics, University of Oxford, Oxford, UK

**Keywords:** Chronic kidney disease, Genome-wide association studies, Hypertension, Chronic kidney disease, Animal physiology

## Abstract

Hypertension (HTN) and chronic kidney disease (CKD) are common in ageing cats. In humans, blood pressure (BP) and renal function are complex heritable traits. We performed the first feline genome-wide association study (GWAS) of quantitative traits systolic BP and creatinine and binary outcomes HTN and CKD, testing 1022 domestic cats with a discovery, replication and meta-analysis design. No variants reached experimental significance level in the discovery stage for any phenotype. Follow up of the top 9 variants for creatinine and 5 for systolic BP, one SNP reached experimental-wide significance for association with creatinine in the combined meta-analysis (chrD1.10258177; *P* = 1.34 × 10^–6^). Exploratory genetic risk score (GRS) analyses were performed. Within the discovery sample, GRS of top SNPs from the BP and creatinine GWAS show strong association with HTN and CKD but did not validate in independent replication samples. A GRS including SNPs corresponding to human CKD genes was not significant in an independent subset of cats. Gene-set enrichment and pathway-based analysis (GSEA) was performed for both quantitative phenotypes, with 30 enriched pathways with creatinine. Our results support the utility of GWASs and GSEA for genetic discovery of complex traits in cats, with the caveat of our findings requiring validation.

## Introduction

Chronic kidney disease (CKD) and systemic hypertension (HTN) are common in the ageing feline population. CKD is reported to affect up to 80% of cats > 15 years when early non-azotemic disease is included and approximately 30% of cats with CKD will be simultaneously diagnosed with HTN^1,2^. However, idiopathic HTN is also recognised in approximately 20% of cats > 9 years^3^. Both CKD and systemic HTN are considered as complex disorders affected by genetic, environmental and lifestyle factors although information in feline medicine is currently limited.

The most common renal pathology identified in cats as they age is tubulointerstitial nephritis with factors such as proteinuria, hypoxia (low packed cell volume), mineral and bone disorder or lifestyle factors (e.g. vaccination and dental disease) implicated in either disease development or progression^4–9^. As in humans, blood pressure (BP) increases with age in the cat, even without predisposing disease conditions^10,11^. However, inter-species difference exists, with the cat showing a marked response to drugs such as the calcium channel blocker amlodipine besylate when compared to humans^12,13^. Comparative analysis may therefore be of interest.

In humans, genome wide association studies (GWAS) have been used to explore in a hypothesis free manner, genetic associations with renal function, CKD, BP and HTN. Translational information from causative or mechanistic genes may enhance either the diagnostic or therapeutic options for patients^14–18^. The first GWAS of HTN was a case–control study in 2007; it failed to identify any genetic associations, subsequently analyses have focused on BP as a quantitative trait and GWASs have achieved much greater statistical power and discovery yield^19,20^. Large scale genetic association analyses have identified over 1,000 associated signals demonstrating that BP is highly polygenic^19,21–29^. Studies in humans have also explored phenotypic outcomes such as CKD (e.g. creatinine-based estimates of Glomerular Filtration Rate creatinine eGFRcreat < 60 ml/min/1.73m^2^), marked reduction in eGFR (e.g. < 45 ml/min/1.73m^2^), end stage renal disease or either incident or progressive decline in renal function^30–34^. Quantitative studies evaluating renal function have identified over 300 loci associated with eGFR explaining approximately 7% of eGFR variance but relatively few loci have translated directly to causal genes and molecular mechanisms by genetic association^35–39^.

There is limited knowledge on genetic risk factors for renal function, CKD, BP or HTN in the cat. Studies have identified a mutation in *PKD1,* encoding for polycystin 1, a cation channel protein which results in feline polycystic kidney disease^40^. Variants in *UMOD*, encoding for uromodulin a highly conserved glycoprotein, have been explored given its potential as a candidate gene for both renal function and BP and previous associations in human medicine^33,36,38,41–43^. To date, monogenic forms of HTN have not been recognised in the cat although, it is possible they exist. Heritability of biological parameters is poorly described in the feline literature; heritability of creatinine using a colony of related cats has been estimated at 25% but SBP has not been explored^44^.

Since the introduction of a feline genotyping array (Feline Illumina Infinium Array) which characterises ~ 63 000 variants across the feline genome, GWAS have become possible^45,46^. Published feline GWAS have focused on rare pedigree related conditions where clear phenotypic cases and controls are available^47–51^. Such studies have successfully identified monogenic mutations resulting in profound phenotypic change. Despite potential limitations, feline genotyping arrays provide the opportunity to consider both monogenic and complex disease traits.

The aims of this study were to perform a GWAS for renal and BP traits, to perform exploratory genetic risk score (GRS) analyses, an approach that has previously been adopted in human medicine to perform more statistically powerful analyses of all genetic loci identified from GWAS combined into an aggregated score in order to test with disease outcomes and to undertake a gene-set enrichment and pathway-based analysis (GSEA)^19,35^.

**Figure 1:**
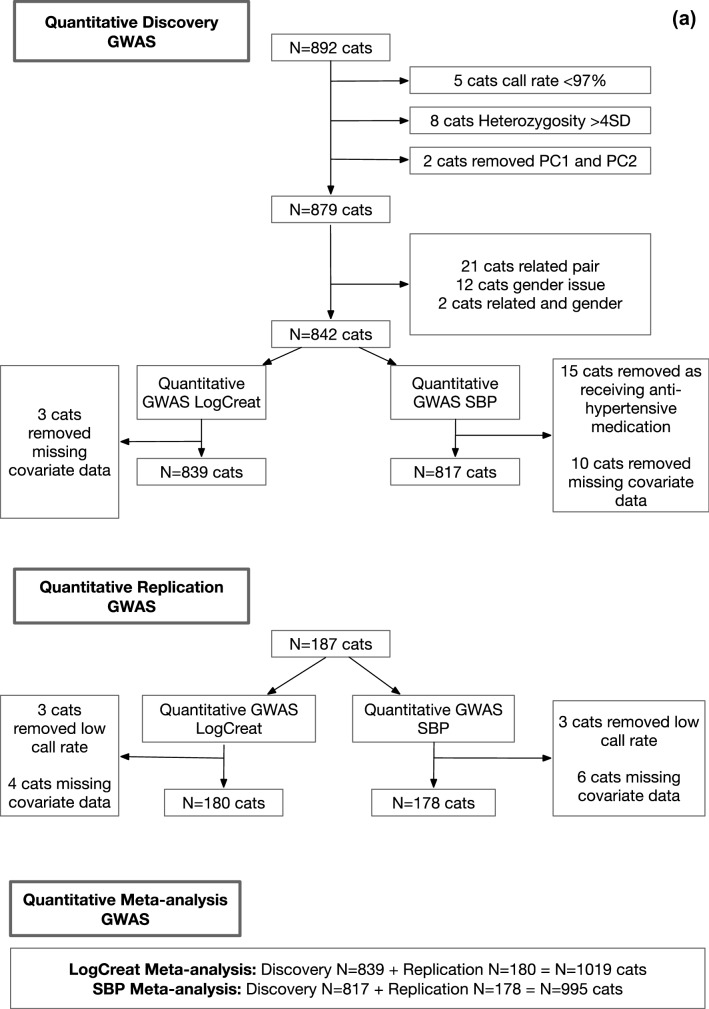

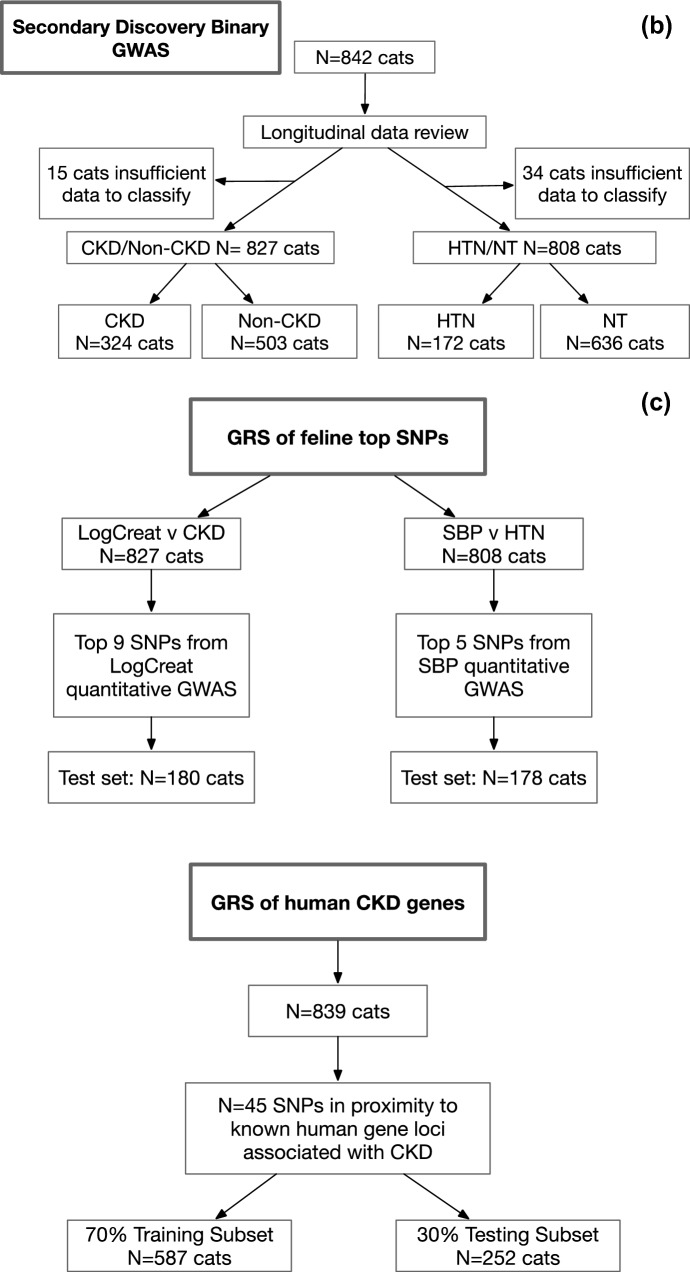
(**a**) Illustration of study design for primary quantitative GWAS. Illustration of discovery and replication design of primary quantitative GWAS for Log creatinine (LogCreat) and systolic blood pressure (SBP). N; number, SD; standard deviation, PC1; principle component 1, PC2; principle component 2, QC; quality control. (**b**) Illustration of study design for secondary binary GWAS. Illustration of discovery GWAS for the binary outcomes chronic kidney disease (CKD) versus non-CKD and hypertension (HTN) versus normotension (NT), N; number. (**c**): Illustration of study design for GRS analyses. The first GRS evaluates the top SNPs from the discovery GWAS for each of LogCreat and SBP, in an attempt to validate the genetic contribution of these SNPs to both SBP and HTN and to LogCreat & CKD within the independent replication sample of cats. The second GRS is based on known human genes and attempts to investigate whether genes known to be associated with human CKD are also associated with renal function in cats. N; number, LogCreat; Log creatinine, CKD; chronic kidney disease, SBP; systolic blood pressure, HTN; hypertension.

A primary GWAS analysis was conducted for the quantitative traits SBP and creatinine within a discovery cohort of 842 cats. Any SNPs of interest (*P* < 2 × 10^–6^) from the quantitative trait discovery analyses were followed up in a replication cohort of 180 cats. Subsequently a meta-analysis GWAS, combining data from the discovery and replication stages together into the total maximum sample size, was performed (Fig. 1a). A secondary GWAS analysis using binary outcomes (CKD and HTN) was also performed (Fig. 1b). Whilst compromising on statistical power, these case–control GWAS analyses provide the opportunity for assessment of clinical outcomes, particularly given the novelty of this study. We also performed two exploratory GRS analyses (Fig. 1c). Firstly, we assessed whether GRSs comprising multiple genetic variants identified from the GWAS of SBP and creatinine are associated with the HTN and CKD status respectively in cats. The second analysis explored whether a GRS comprising variants in genes associated with kidney disease in humans was associated with renal traits in cats. Finally, a GSEA was performed using GWAS data for both quantitative traits of SBP and renal function.

## Results

### Clinical case description

The discovery GWAS cohort comprised 842 domestic shorthair and longhair cats with a median age of 13.3 years (11.0, 15.6) and the replication stage included 180 cats (median age 13.6 years (10.1, 15.8). These groups were combined for GWAS meta-analysis. Comparisons of clinical data for cats included in the evaluation of the quantitative traits creatinine and systolic BP are provided in Tables 1 and 2 respectively. There were statistically significant differences in some clinicopathological variables between cats from the discovery versus replication stages including lower creatinine, potassium, packed cell volume, SBP, urine specific gravity and total T4 concentrations, although small enough to not be deemed clinically significant. Cats included in the discovery stage GWAS for quantitative traits were reviewed for inclusion in the secondary binary outcome GWAS for CKD (N = 827; CKD N = 324, Non-CKD N = 503) and HTN (N = 808; HTN N = 172, normotension (NT) N = 636).

**Table 1:** Baseline signalment and clinicopathological data for cats included in the quantitative association with Log creatinine in the discovery and replication stages.

Variable	Discovery stage		Replication stage		*p*
	Median (25th, 75th percentile)	Number (n)	Median (25th, 75th percentile)	Number (n)	
Age (years)	13.3 (11.0, 15.6)	839	13.6 (10.1, 15.8)	187	0.488
Sex (by computerised database)	Female entire Female neutered Male entire Male neutered	7 416 6 410	Female entire Female neutered Male entire Male neutered	3 104 2 78	
Breed	Domestic longhair Domestic shorthair	108 731	Domestic longhair Domestic shorthair	27 160	0.281
Body weight (kg)	3.95 (3.29, 4.75)	816	3.92 (3.17, 4.60)	186	**0.016**
Creatinine (µmol/l)	154.3 (125.9, 194.0)	839	144.5 (120.4, 183.0)	187	0.414
Phosphorus (mmol/l)	1.32 (1.13, 1.55)	838	1.25 (1.09, 1.45)	187	**0.01**
Potassium (mmol/l)	4.00 (3.73, 4.35)	832	3.96 (3.72, 4.20)	186	**0.015**
Packed cell volume (%)	35 (32, 39)	828	35 (32, 39)	184	0.413
Systolic blood pressure (mmHg)	136.8 (121.2, 154.0)	839	131.3 (115.2, 146.7)	187	**0.008**
Urine specific gravity	1.030 (1.019, 1.046)	573	1.024 (1.018, 1.038)	112	**0.009**
Total Thyroxine (nmol/l)	19.9 (13.5, 26.3)	839	19.8 (13.5, 24.1)	187	0.371

*P*; statistical significance comparing baseline parameters for discovery and replication stage for cats included in quantitative association analysis with LogCreat (P-values in bold if *P * < 0.05).

**Table 2:** Baseline signalment and clinicopathological data for cats included in the quantitative association with systolic blood pressure from the discovery stage with comparison to replication analysis.

Variable	Discovery Stage		Replication Stage		*p*
	Median (25th, 75th percentile)	Number (n)	Median (25th, 75th percentile)	Number (n)	
Age (years)	13.3 (11.0, 15.6)	817	13.6 (10.1, 15.8)	187	0.542
Sex (by computerised database)	Female entire Female neutered Male entire Male neutered	6 405 6 400	Female entire Female neutered Male entire Male neutered	3 104 2 78	–
Breed	Domestic longhair Domestic shorthair	105 712	Domestic longhair Domestic shorthair	27 160	–
Body weight (kg)	3.96 (3.29, 4.75)	795	3.92 (3.17, 4.60)	186	0.296
Creatinine (µmol/l)	154.0 (125.9, 193.6)	817	144.5 (120.4, 183.0)	187	**0.019**
Phosphorus (mmol/l)	1.32 (1.13, 1.55)	816	1.25 (1.09, 1.45)	187	**0.012**
Potassium (mmol/l)	4.00 (3.8, 4.36)	817	3.96 (3.72, 4.20)	186	**0.01**
Packed cell volume (%)	35 (32, 39)	806	35 (32, 39)	184	0.373
Systolic blood pressure (mmHg)	136.4 (121.0, 153.6)	817	131.3 (115.2, 146.7)	187	**0.013**
Urine specific gravity	1.031 (1.019, 1.046)	556	1.024 (1.018, 1.038)	112	**0.007**
Total Thyroxine (nmol/l)	19.8 (13.5, 26.3)	817	19.8 (13.5, 24.1)	187	0.470

*P*; statistical significance comparing baseline parameters for discovery and replication stage for cats included in quantitative association analysis with systolic blood pressure. Replication data are found in Table 1. (*P*-values in bold if* P* < 0.05).

### Primary analysis: discovery stage GWAS for quantitative traits

**Figure 2:**
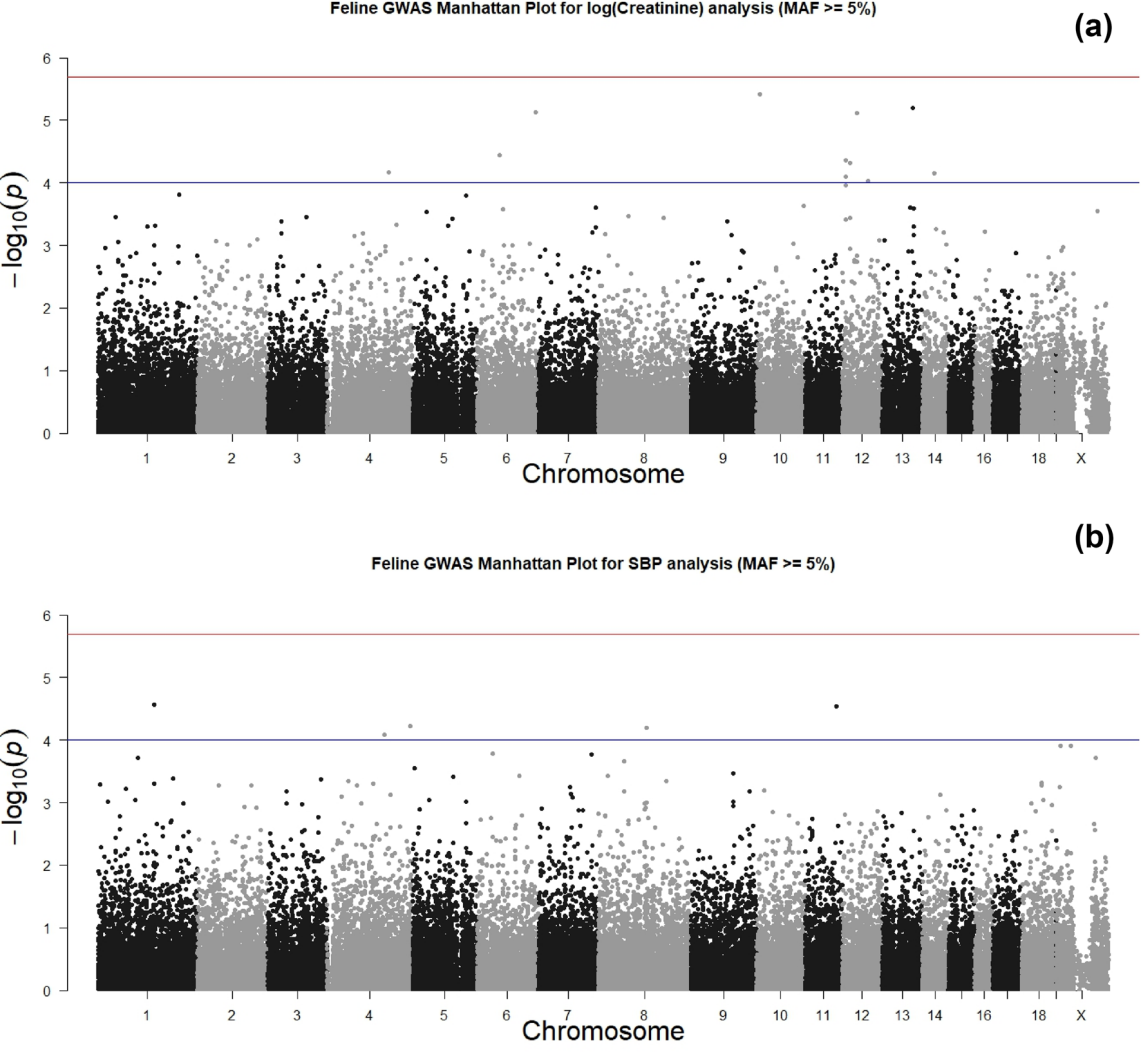
(**a**) Manhattan plot for GWAS evaluating Log creatinine as a quantitative trait in the discovery stage. Manhattan plot of the discovery genome-wide association study in 839 cats. The y axis shows the −log10* P* values of SNPs with MAF ≥ 5% and the x axis shows their chromosomal positions. Horizontal blue and red lines represent the thresholds of* P* = 1 × 10^–4^ used for selecting follow-up SNPs for replication and* P* = 2 × 10^–6^ denoting experimental-wide significance, respectively. No SNPs reached experimental-wide significance in the discovery stage. (**b**): Manhattan plot for GWAS evaluating systolic blood pressure as a quantitative trait in the discovery stage. Manhattan plot of the discovery genome-wide association study in 817 cats. The y axis shows the -log10 P values of SNPs with MAF ≥ 5% and the x axis shows their chromosomal positions. Horizontal blue and red lines represent the thresholds of* P* = 1 × 10^–4^ used for selecting follow-up SNPs for replication and *P* = 2 × 10^–6^ denoting experimental-wide significance, respectively. No SNPs reached experimental-wide significance in the discovery stage.

In the GWAS discovery analysis for Log Creatinine (LogCreat), analysing 839 cats, no SNPs reached experimental wide significance (*P* < 2 × 10^–6^), with the minimum *P*-value being *P* = 3.85 × 10^–6^ (Table 3, Fig. 2a). Similarly, in the GWAS discovery analysis for SBP, analysing 817 cats, no SNPs reached experimental wide significance, with the minimum *P*-value being *P* = 7.48 × 10^–5^ (Table 4, Fig. 2b).

**Table 3:** Comparison of discovery, replication and meta-analysis for Log creatinine.

SNP	Discovery										
	CHR	BP	A1	A2	N_disc	HWE P_disc	Freq1_disc	BETA_disc	SE_disc	P_disc	N_rep
**chrD1.10258177**	**D1**	**8,482,421**	**G**	**A**	**839**	**0.163**	**0.08**	**0.14**	**0.03**	**3.85 × 10**^**–06**^	**180**
**chrD4.72377931**	**D4**	**73,436,741**	**A**	**G**	**839**	**0.340**	**0.12**	**0.12**	**0.03**	**6.35 × 10**^**–06**^	**180**
**chrE1.56119546**	**E1**	**29,657,901**	**A**	**G**	**837**	**0.136**	**0.38**	**0.07**	**0.02**	**7.06 × 10**^**–05**^	**180**
**chrB1.176960684**	**B1**	**147,421,352**	**A**	**G**	**839**	**0.459**	**0.11**	**-0.108**	**0.03**	**6.80 × 10**^**–05**^	**114**
**chrD3.10545751**	**D3**	**8,222,044**	**G**	**A**	**839**	**0.069**	**0.11**	**− 0.11**	**0.03**	**4.31 × 10**^**–05**^	**180**
chrUn26.3715257	D3	17,723,307	G	A	839	0.530	0.16	0.09	0.02	4.80 × 10^–05^	180
chrB3.69084516	B3	55,418,968	G	A	839	0.451	0.10	0.12	0.03	3.60 × 10^–05^	180
chrD3.51239128	D3	35,001,715	G	A	839	0.626	0.28	0.08	0.02	7.56 × 10^–06^	180
chrD3.84193125	D3	61,072,237	G	A	839	0.09	0.27	0.07	0.02	9.34 × 10^–05^	180

SNP	Replication					Meta− Analysis					
	HWE P_rep	Freq1_rep	BETA_rep	SE_rep	P_rep	Freq1_meta	BETA_meta	SE_meta	P_meta	P_Het_Meta	Direction of effect (discovery to meta)
**chrD1.10258177**	**0.087**	**0.054**	**0.11**	**0.08**	**0.1704**	**0.07**	**0.14**	**0.03**	**1.34 × 10**^**–06**^	**0.706**	**++ **
**chrD4.72377931**	**0.641**	**0.09**	**0.05**	**0.07**	**0.4944**	**0.12**	**0.11**	**0.02**	**7.12 × 10**^**–06**^	**0.331**	**++ **
**chrE1.56119546**	**0.286**	**0.41**	**0.05**	**0.04**	**0.202**	**0.39**	**0.07**	**0.02**	**2.93 × 10**^**–05**^	**0.722**	** + + **
**chrB1.176960684**	**0.646**	**0.11**	**− 0.08**	**0.07**	**0.2444**	**0.11**	**− 0.10**	**0.03**	**3.20 × 10**^**–05**^	**0.754**	**–**
**chrD3.10545751**	**0.320**	**0.12**	**− 0.04**	**0.06**	**0.5347**	**0.12**	**− 0.09**	**0.02**	**6.07 × 10**^**–05**^	**0.270**	**–**
chrUn26.3715257	0.116	0.17	− 0.01	0.05	0.8575	0.16	0.08	0.02	0.0002124	0.082	+ −
chrB3.69084516	0.473	0.11	− 0.02	0.06	0.7522	0.10	0.09	0.03	0.0002838	0.041	+−
chrD3.51239128	0.240	0.25	− 0.03	0.04	0.5218	0.28	0.07	0.02	0.0001066	0.017	+
chrD3.84193125	0.459	0.27	− 0.03	0.04	0.4481	0.27	0.06	0.02	0.001061	0.022	+−

SNP; single nucleotide polymorphism (Named from Illumina Feline Infinium Array), CHR; chromosome (FelCat5; Felis_catus-6.2 Genome assembly), BP; base pairs (FelCat5; Felis_catus-6.2 Genome assembly), A1; minor allele, A2; major allele, N; number, HWE; Hardy Weinberg Equilibrium, freq1; frequency of minor allele, BETA; beta, SE; standard error, p; significance, _disc; discovery, _rep; replication, _meta; combined metanalysis.

Data table ordered by meta-analysis significance. Bold indicates experimental wide significance for meta-analysis (*P* < 1 × 10^–6^) and/or concordance of direction of effect.

**Table 4:** Comparison of discovery, replication and meta-analysis for systolic blood pressure.

SNP			Discovery							
	CHR	BP	A1	A2	N_disc	HWE P_disc	Freq1_disc	BETA_disc	SE_disc	P_disc
**chrA1.175695892**	**1**	**134,277,731**	**A**	**G**	**817**	**0.874**	**0.30**	**6.24**	**1.48**	**2.68 × 10**^**–5**^
chrD2.99141949	11	74,668,392	A	G	812	0.155	0.38	5.74	1.37	2.93 × 10^–5^
chrB1.225124311	4	200,288,643	A	C	817	0.582	0.24	6.27	1.56	6.00 × 10^–5^
chrC1.128491506	8	114,624,871	G	A	817	0.480	0.21	− 6.71	1.67	6.33 × 10^–5^
chrB1.167814582	4	136,873,181	G	A	817	0.390	0.29	− 5.95	1.50	8.19 × 10^–5^

SNP			Replication							
	CHR	BP	N_rep	HWE P_rep	Freq1_rep	BETA_rep	SE_rep	P_rep		
**chrA1.175695892**	**1**	**134,277,731**	**178**	**0.066**	**0.33**	**1.17**	**2.99**	**0.6951**		
chrD2.99141949	11	74,668,392	178	0.879	0.41	− 0.67	3.07	0.8265		
chrB1.225124311	4	200,288,643	175	4.9 × 10^^− 22^	0.11	− 0.48	3.58	0.8933		
chrC1.128491506	8	114,624,871	178	0.022	0.23	0.71	3.32	0.8304		
chrB1.167814582	4	136,873,181	178	0.854	0.27	4.08	3.54	0.2512		

SNP			Meta− Analysis							
	CHR	BP	Freq1_meta	BETA_meta	SE_meta	P_meta	P_Het_Meta	Direction of effect (discovery to meta)		
**chrA1.175695892**	**1**	**134,277,731**	**0.31**	**5.24**	**1.32**	**7.48 × 10**^**–5**^	**0.129**	**++ **		
chrD2.99141949	11	74,668,392	0.39	4.68	1.25	0.0001771	0.056	+−		
chrB1.225124311	4	200,288,643	NA	NA	NA	NA	NA	NA		
chrC1.128491506	8	114,624,871	0.21	− 5.21	1.49	0.0004715	0.046	−+		
chrB1.167814582	4	136,873,181	0.29	− 4.42	1.38	0.001404	0.009	−+		

SNP; single nucleotide polymorphism (Named from Illumina Feline Infinium Array), CHR; chromosome (FelCat5; Felis_catus-6.2 Genome assembly), BP; base pairs (FelCat5; Felis_catus-6.2 Genome assembly), A1; minor allele, A2; major allele, n; number, HWE; Hardy Weinberg Equilibrium, disc; discovery cohort, rep; replication cohort, meta; meta-analysis, freq1; frequency of minor allele, SE; standard error, *p*; significance. Bold indicates experimental wide significance for meta-analysis (*P* < 1 × 10^–6^) and/or concordance of direction of effect.

### Primary analysis: replication study and meta-analysis for quantitative traits

Nine SNPs with *P* < 1 × 10^–4^ were taken forward from the discovery to the replication stage for LogCreat in 180 cats (Table 3) and 5 SNPs with *P* < 10^–4^ for SBP in 178 cats (Table 4). HWE was not demonstrated for one SNP in the replication phase for SBP (chrB1.225124311). No SNPs reached Bonferroni-adjusted significance level (*P* < 0.05/9 = 0.0056 for LogCreat; *P* < 0.05/4 = 0.0125 for SBP) in the replication analyses, hence no SNPs formally replicated (Tables 3 and 4).

The discovery and replication data were meta-analysed together (9 SNPs for LogCreat and 4 SNPs for SBP) and there was no evidence of any heterogeneity in the effect estimates between discovery and replication (Meta_Het_*P* > 0.01; Tables 3 and 4). One SNP (chrD1.10258177) reached experimental wide significance (*P* = 1.34 × 10^–6^) in the combined meta-analysis for LogCreat (N = 1,019; Table 3) with concordant direction of effect between discovery and replication stages. For this SNP chrD1.10258177, cats carrying the G allele resulted in a 0.14 increase in LogCreat (standard error (SE); 0.03) per unit allele increase (Table 3). Searching 1 Mbp up and downstream from this SNP we identified 8 predicted genes and 3 predicted open reading frames:*, ZC3H12C, RDX, FDX1, ARHGAP20, COLCA2, POU2AF1, BTG4, LAYN and C11orf87, C11orf53, C11orf88*.

No SNPs reached experimental wide significance (*P* < 2 × 10^–6^) for SBP despite the larger sample size when combining discovery and replication cohorts (N = 995; Table 4) and between the discovery and replication stages only one SNP had a concordant direction of effect.

### Secondary analysis: Discovery stage GWAS of binary traits

No SNPs reached experimental wide significance in the GWAS analyses for CKD (N = 324) versus non-CKD (N = 503; Supplementary Table S1, Supplementary Fig S4) or HTN (N = 172) versus NT (N = 636; Supplementary Table S2, Supplementary Fig S5), with the minimum *P*-value being *P* = 5.82 × 10^–5^ for association with CKD and *P* = 4.15 × 10^–5^ for HTN/NT (Supplementary Table S3). Bivariate plots of the *P*-values from the GWAS discovery and binary trait analyses summary statistics were produced (Supplemental Fig S6 and Fig S7). For both LogCreat/CKD and SBP/HTN there was a significant positive correlation between the quantitative trait analyses and the binary disease analysis (r^2^ = 0.26, *P* < 2 × 10^–16^ for SBP/HTN and r^2^ = 0.45, *P* < 2 × 10^–16^ LogCreat/CKD), showing good concordance in the results, despite an overall lack of power for the single-SNP analyses. SNP chrD1.10258177 with a significant association for LogCreat shows some suggestive level of association for the binary outcome CKD (*P* = 0.0001 and OR 2.057).

### Genetic risk score analyses

The first exploratory GRS analysis, which comprised multiple genetic variants identified from the GWAS of SBP and LogCreat, was tested for association with HTN (N = 808) and CKD (N = 827) status, using cats from the discovery and replication GWAS stages as the discovery and testing datasets respectively. As initial proof of concept, we confirmed, within the same discovery sample, that these aggregated scores of top SNPs from the quantitative traits were strongly associated with the clinical disease outcomes: the SBP-GRS was significantly associated (*P* = 9.4 × 10^–3^) with increased risk of HTN (N = 808); and the Logcreat-GRS was significantly associated (*P* = 6.1 × 10^–14^) with increased risk of CKD (N = 827). However, testing of the GRS in the independent replication sample of cats (N = 180) indicated no significant results: SBP-GRS with SBP (*P* = 0.606) or HTN (*P* = 0.926); and the Logcreat-GRS with either LogCreat (*P* = 0.599) or CKD (*P* = 0.266).

Our second exploratory GRS analysis tested a human CKD-GRS created using the closest feline chip SNPs corresponding to 45 known CKD-associated genes. The discovery cohort was randomly divided into a 70% training subset (n = 587) and a 30% testing subset (n = 252).

The results of the GRS analysis were non-significant: the GRS was not associated with LogCreat as a continuous variable (*P* = 0.986) and did not show any significant difference (*P* = 0.788) in the levels of LogCreat from the quintiles analysis between the cats within the top 20% of the GRS risk distribution vs. the cats in the bottom 20%. We note though from the single-SNP results that only 6 of these 45 SNPs showed nominal significance (*P* < 0.05) individually within the 70% training data (Supplementary Table S4).

### Gene-set enrichment and pathway-based analysis (GSEA)

**Figure 3:**
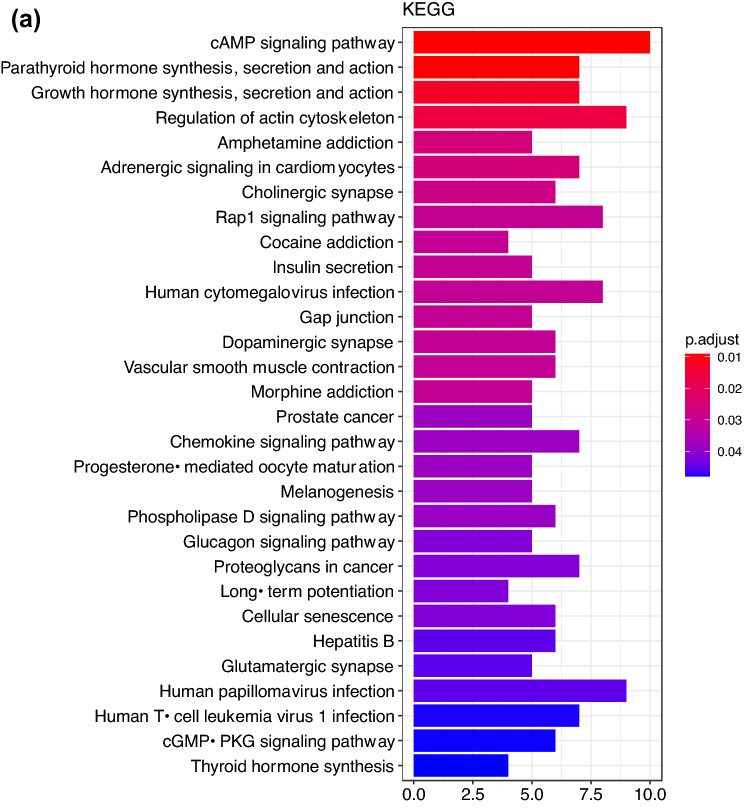

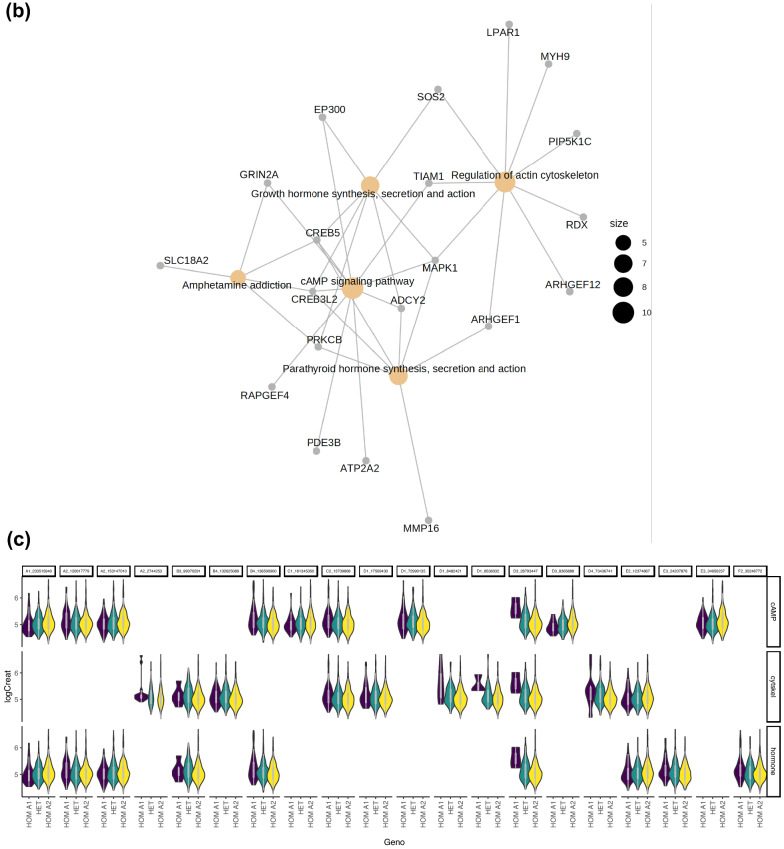

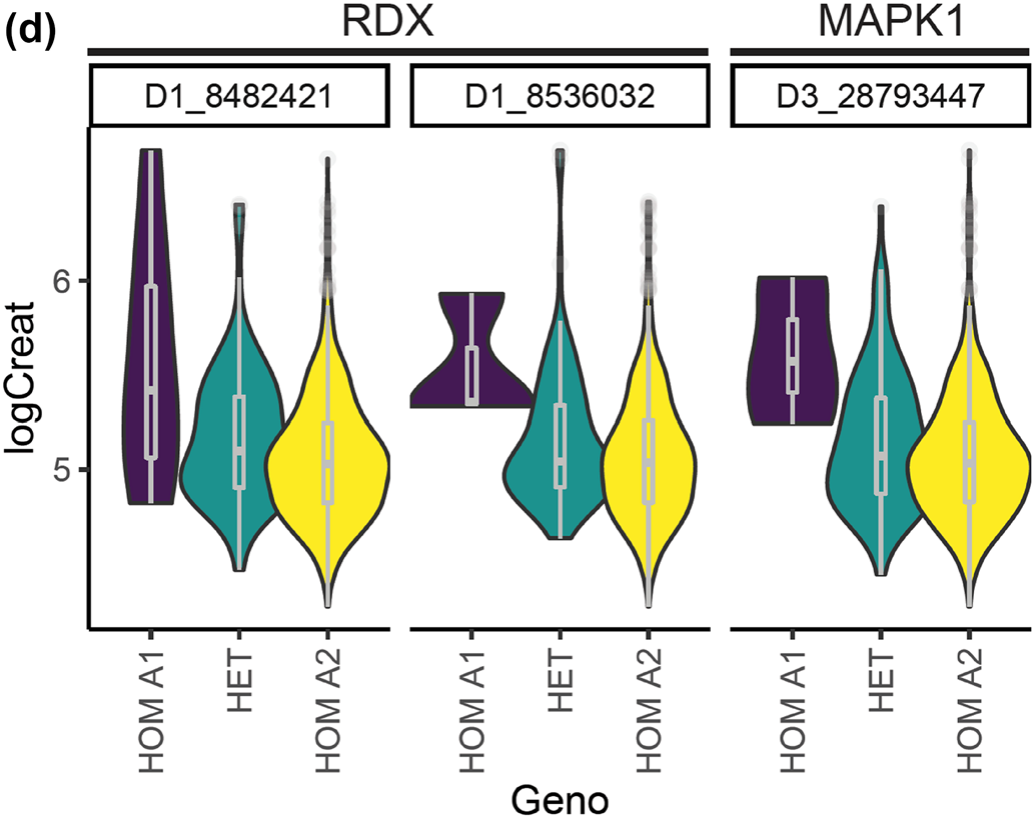
Gene set enrichment analysis of Log Creatinine. (**a**) Bar plot showing enriched KEGG pathways for quantitative renal trait Log Creatinine. Results of gene-set enrichment analysis (GSEA): Bar charts of significantly enriched KEGG pathways. Number of genes (n) corresponding to nominally associated LogCreat GWAS SNPs is represented on the x-axis with adjusted significance (*P* < 0.05) indicated by order and colour trend. (**b**) Gene network plot for enriched KEGG pathways associated with the renal trait Log creatinine. Gene network plot showing identified feline genes (grey nodes) and overlap between enriched KEGG pathways for renal trait Log creatinine. Gold nodes represent top 5 enriched KEGG pathways including cAMP signalling, parathyroid hormone synthesis, secretion and action, growth hormone synthesis, secretion and action, regulation of actin cytoskeleton and amphetamine addiction where nodal size is proportional to the number of nominally associated LogCreat GWAS genes in each pathway. (**c**): Violin plots showing Log Creatinine by genotype for SNPs corresponding to genes of the top 4 pathways enriched in GSEA. cAMP; cyclic adenosine monophosphate pathway, cytskel; cytoskeleton pathway, hormone; parathyroid hormone and growth hormone pathways, HOM A1; homozygous allele 1, HOM A2; homozygous allele 2, HET; heterozygous, LogCreat; Log creatine. (**d**) Violin plots of Log Creatinine by genotype for the most striking SNPs corresponding to RDX and MAPK1 from top enriched GSEA pathways. HOM A1; homozygous allele 1, HOM A2; homozygous allele 2, HET; heterozygous, LogCreat; Log creatinine.

GWAS was complemented with a GSEA exploring gene set enriched pathways associated with the quantitative traits LogCreat and SBP. GWAS SNPs demonstrating nominal (*P* < 0.01*)* association with LogCreat and SBP were annotated with ENSEMBL FelCat5 genes when located in or within 5 Kb flanking the gene boundaries. For each trait, the subset of these genes found in both the discovery and replication cohort were included in the GSEA. The GSEA for LogCreat identified 30 enriched KEGG pathways corresponding with 33 unique GWAS genes (*P* < 0.05; Fig. 3a, Supplementary Table S5). The top pathways are of particular interest and include cyclic adenosine monophosphate (cAMP) signalling, parathyroid hormone (PTH) synthesis, secretion and action, growth hormone (GH) synthesis, secretion, action and regulation of actin cytoskeleton pathways (Table 5; Fig. 3b–d). GSEA showed no evidence of pathway enrichment for the quantitative trait SBP.

**Table 5:** Top 4 enriched KEGG pathways associated with quantitative renal trait Log Creatinine.

Term ID	Description	Count	P value	Genes
hsa04024	cAMP signalling pathway	10/81	0.009178	ADCY2/PDE3B/ATP2A2/MAPK1/GRIN2A/CREB5/CREB3L2/EP300/RAPGEF4/TIAM1
hsa04928	Parathyroid hormone synthesis, secretion and action	7/81	0.009178	ADCY2/MAPK1/ARHGEF1/PRKCB/MMP16/CREB5/CREB3L2
hsa04935	Growth hormone synthesis, secretion and action	7/81	0.012676	ADCY2/MAPK1/PRKCB/CREB5/CREB3L2/SOS2/EP300
hsa04810	Regulation of actin cytoskeleton	9/81	0.016506	RDX/ARHGEF12/MAPK1/LPAR1/ARHGEF1/PIP5K1C/SOS2/MYH9/TIAM1

Term ID; KEGG pathway identification, Description; descriptor of enriched KEGG pathway, Count; number of feline genes enriched within given KEGG pathway, %; percentage of feline genes compared to genes in KEGG pathway, P value; significance, Genes; feline genes associated with enriched KEGG pathway.

## Discussion

This is the first feline GWAS to investigate complex disease traits in domestic cats. Although no SNPs reached significance in the separate discovery or replication GWAS stages for either SBP or LogCreat, a single SNP chrD1.10258177 reached experimental-wide significance, with concordant direction of effect, in the combined meta-analysis for association with LogCreat. It is impossible to draw strong conclusions, however, there were a few genes of potential interest from a pathophysiological perspective within 1Mbp of this locus. *ZC3H12C* encoding for zinc finger CCCH type containing C12, plays a role controlling macrophage activation, inhibits production of TNF-alpha and inducible nitric-oxide synthase promotor activity^52^ and in vitro, inhibits the endothelial inflammatory response^53^. CKD is widely considered an inflammatory disease and as such altered expression of genes with a negative regulatory response could contribute to the progression of disease. *RDX* encodes for radixin, a member of the ERM (ezrin-radixin-moesin) proteins which provide cellular structure, linking the plasma membrane to the cystoskeleton and providing a mechanism for regulation of signal transduction pathways^54–56^. The role of radixin in CKD has not been explored although other ERM proteins have been associated with regulation of fibrosis^57,58^, *FDX1* encoding for Ferrodoxin 1 is a small iron sulfur protein important for electron transfer particularly to cytochrome P450 enzymes and iron homeostasis and has also been implicated in Vitamin D homeostasis via CYP enzymes^59,60^. Finally, *LAYN* encoding for layilin is a transmembrane c-type lectin-homologous protein which has been associated in vitro with TNF-alpha induced epithelial to mesenchymal transformation of renal tubular epithelial cells from patients with IgA nephropathy, therefore potentially playing a role in renal disease progression and fibrosis^61.^.

As part of the gene mapping performed (Supplemental Methods), none of these predicted genes have previously been identified from human GWAS exploring renal function traits^35^. However, evaluating these genes in the GWAS catalog revealed that in humans the *RDX/ZC3H12C* locus has previously been associated with PTH concentration in women and also with high density lipoprotein (HDL) cholesterol in human GWAS^62–64^. In humans, there is an association among dyslipidemia, atherosclerosis, cardiovascular and renal disease although species differences mean that atherosclerosis is a rare phenomenon in cats^64,65^. The *FDX1/ARHGAP20* locus has been associated with renal sinus fat which has a proposed link between obesity and renal function^66–69^. Renal pelvis fat can be identified during the imaging of feline kidneys but has never been explored as a risk factor for CKD^70^. Finally, the *POU2AF1* locus has been associated with both nephrolithiasis and urinary pH which are current areas of interest in feline CKD^71–73^.

To increase statistical power beyond single-SNP analyses, GRS analyses were performed. GRS from the discovery sample showed that the aggregated GRS of the top follow-up SNPs from the SBP and LogCreat were significantly associated with the binary clinical outcomes of HTN and CKD, respectively. However, insufficient power remains one of the reasons why it was not possible to independently validate the association of our top discovery SNPs for SBP or LogCreat with either SBP or HTN, or with LogCreat or CKD, respectively. This is perhaps unsurprising, given that, due to the limited replication sample size available, none of the top discovery SNPs formally replicated individually in the primary analyses and very few even had concordant direction of effect between the discovery and replication data.

Our second exploratory GRS analysis attempted to investigate whether genes known to be associated with CKD in humans may also play a role in influencing renal function and creatinine levels in cats. Unfortunately, our GRS including the closest feline SNP to a set of 45 human CKD associated genes did not show any evidence of association with LogCreat. We are therefore unable to demonstrate from our data that genes associated with renal function in humans are also associated with renal function in cats. However, due to the small sample sizes, the limitations in predicted gene location using the currently available feline genome data and also not knowing the exact causative genes in humans, this does not rule out the future potential of translation between humans and cats.

GSEA identified 4 KEGG pathways of interest in relation to renal function; cAMP, PTH, GH and actin cytoskeleton. Of particular note, was the identification of *RDX* from the cystoskeleton pathway which had previously been identified in proximity to SNP chrD1.10258177 from the LogCreat GWAS. Identification of the actin cytoskeleton pathway raises the potential importance of maintenance of both podocyte and tubular structure in the pathogenesis of CKD. cAMP is a universal second messenger found in cells of all biological systems with many physiological roles within the kidney^74^. In relation to tubulointerstitial nephritis, increased cAMP exerts anti-fibrotic effects and cyclic nucleotide modulation is a potential therapeutic target for renal fibrosis^75,76^. PTH is an important phosphoregulatory hormone contributing to the development of renal mineral and bone disorder^77^. GH and insulin-like growth factor (IGF) are important not only for the physiological development of the kidneys but also in renal homeostasis with the inflammatory state of CKD potentially altering the GH-IGF axis^78,79^.

This study has highlighted some of the challenges associated with the exploration of complex genetic traits and disease conditions currently in the domestic cat. Inability to identify significant loci in the discovery stage can be attributed to small sample size and insufficient power for discovery. Power calculations were performed for study design using data from a small extreme phenotype pilot study (50 severely HTN and 50 NT cats). Due to the novelty of the feline GWAS array and no prior analysis of BP and renal traits in cats, calculations were based on assumptions for the heritability and LD structure from human GWAS, with expected effect estimates based on the current knowledge of effect sizes of SNPs on SBP and renal function in human medicine. Despite this being one of the largest feline genetic studies, it is very small compared to human GWAS which frequently include several hundred thousand individuals in the discovery stage and more recently up to 1 million individuals for meta-analyses^19,35^. Studies exploring the diagnosis and management of CKD in the UK indicate that 96.1% of cats are cross-breeds rather than pedigree cats, emphasising the importance of studying common disease within the crossbreed population^80^. Future studies of similar complex traits in domestic cats should therefore aim to test sample sizes of at least 1,000 as the very minimum required.

Ideally, we would use heritability analyses as the ideal approach to formally confirm that genetics play a role in BP regulation and renal traits in cats. Previous exploration of heritability using a colony of cats estimated the heritability of creatinine to be 25%^81^. We attempted to calculate the heritability of our BP and renal traits from feline GWAS data using GCTA software^82^, but this proved not to be possible, with no meaningful results obtained, due to both the insufficient array SNP density and the small sample size. We are therefore at least encouraged by the positive proof of concept result from the exploratory GRS analysis, showing some evidence, albeit in the same discovery sample, that the top variants influencing BP and renal traits are also associated with the clinical outcomes of HTN and CKD in cats.

A further limitation of this study is the low density of the feline Illumina Infinium iSelect DNA array for identification of loci associated with complex disease conditions^45,83,84^. SNPs incorporated onto this array have been remapped to the feline genome assembly 6.2^46,49^ and subsequently assembly 8.0 and 9.0^45,85^. The array average marker distance varies based on chromosome and region of the array, with an average marker distance of 37,741 bp although in certain areas gaps of up to 3 Mbp are reported^45^. The lack of other genotyped variants in LD or close proximity to the follow-up SNP of interest makes it challenging to assess if there is wider support for an association signal at a locus. In human studies, most GWAS analyses take advantage of genetic datasets that have been densely imputed from imputation reference panels, which is not yet feasible for feline studies. To date, this feline array has proved to be useful in the identification of traits under selection or recessive traits, e.g. congenital myasthenic syndrome in the Devon Rex^48^ or hypokalemia in the Burmese^86^ or for dominant traits that are under positive selection e.g. Scottish Fold cat folded ears^47^. However, exploration of complex traits is fundamentally vital to veterinary medicine, given that these represent the most common medical conditions that are impacting the health and welfare of the largest number of feline patients. A SNP array with denser coverage is currently under development as part of the Feline 99 Lives Consortium^87^.

Sparsity of variants was also a disadvantage when performing the GSEA given that relatively few SNPs lie within a known gene sequence or flanking region. Overlap of genes within the top pathways gives challenges in terms of determining relevance of over-representation within a given pathway. However, novel genes from each pathway were identified suggesting each may have some independent relevance. It should be recognised that the nominal criteria used for SNP inclusion within this analysis increases the risk of false positive associations being reported but represents a balance between discovery and output for this exploratory analysis. Failure to identify pathways enriched in relation to SBP follows lack of significance identified in the GWAS and most likely relates to this being a complex polygenic trait with extreme limitations in terms of sample size.

The strengths of this study include the unique DNA archive combined with standardised phenotypic evaluation available including biochemical and blood pressure data. In addition to cross-sectional data, a large proportion of this population of cats also had standardised longitudinal data facilitating clinical phenotypic classification. Nevertheless, there are factors which may have influenced the classification of cats. Plasma creatinine can be influenced by biological factors, in particular muscle condition and concurrent disease e.g. hyperthyroidism. Total thyroxine concentrations were reviewed in all cats to exclude hyperthyroidism and cats were excluded if they were receiving drugs that could influence GFR. Careful review of clinical data permitted binary classification, however, it is recognised that cats may demonstrate evidence of CKD prior to the onset of azotemia. Therefore, misclassification as non-CKD when cats may have had International Renal Interest Society (IRIS) stage 1 or early stage 2 CKD is possible^88^. As in humans, situational HTN can influence SBP measurements. BP measurements for all cats were performed in accordance with American College of Veterinary Internal Medicine HTN consensus guidelines, but some degree of inaccuracy is inevitable. Careful longitudinal assessment of SBP was used for classification of cats with HTN with exclusion of cases where insufficient data were available to prevent inappropriate classification. There were statistically significant differences in biochemical parameters between the discovery and replication cohorts. However, at the time of analysis, every genomic DNA sample within the biobank was used for this study and therefore alternative selection of replication stage cats was not possible. Clinically the cats were recruited using the same eligibility criteria and numerically the differences did not raise concern for clinical differences between groups.

This is the first feline GWAS to explore genetic associations with SBP and renal function in cats. Our analysis identified a single SNP with experimental wide significance for the quantitative trait creatinine and highlighted, through GSEA, enriched biological pathways associated with this trait. Further validation work would be required to draw strong conclusions in relation to the specific loci identified. Nevertheless, this study positively supports the utility of GWAS and GSEA in feline medicine, especially if arrays with denser coverage of the feline genome could be developed in the future, and larger studies may also enable further success from GRS analyses of clinical outcomes and the testing of human genes in cats, which we attempted here.

## Methods

Cats > 8 years that had been evaluated as part of a longitudinal elderly cat monitoring programme at the Royal Veterinary College including healthy cats or those with known conditions of interest including CKD and HTN were included in this study (Supplementary Methods). The clinic study protocols received ethical approval by the Royal Veterinary College’s Clinical Research and Ethical Review Board (CRERB; URN: 2013 1258) which included routine storage of cell pellets for genomic DNA (gDNA) extraction. All cats included in this study were client owned and informed consent was obtained prior to enrolment. Clinical data pertaining to each cat included in this study was obtained through standard veterinary care offered by the clinic. All methods were carried out in accordance with the research guidelines at the Royal Veterinary College and are reported in accordance with ARRIVE guidelines.

An initial cohort of cats (n = 842) was used for the discovery GWAS evaluating the quantitative traits SBP and creatinine utilising the feline Illumina array. SNPs of interest (*P* < 2 × 10^–6^) were then taken forwards using a second cohort of cats (n = 187) into a replication and meta-analysis stage. A secondary discovery analysis exploring the binary traits CKD and HTN was performed but due to limited sample sizes replication analyses were not evaluated. Exploratory GRS analyses were performed to increase statistical power beyond single-SNP analyses considering firstly the top SNPs from the quantitative GWAS meta-analyses and secondly testing known human CKD loci for association with feline renal function.

### Eligibility criteria for quantitative genome wide association study discovery and replication stage

Only domestic shorthair (DSH) or long-hair (DLH) cats were included in this study. The first visit with concurrent SBP (Doppler technique) and plasma creatinine concentration (Idexx Laboratories, Wetherby, UK) together with a stored cell pellet for gDNA extraction was selected. Information on inclusion/exclusion criteria is provided in supplemental methods. Signalment, clinical, and laboratory data were extracted for all cats including age, sex, body weight, potassium, phosphorus, packed cell volume and urine specific gravity.

Eligibility criteria for cats included in the replication stage were identical to the discovery stage. Case selection was based on availability of breed, creatinine, SBP data and stored cell pellet for gDNA extraction. Mann Whitney U-tests were used to compare clinical parameters between the discovery and validation cohorts. All available cats that fulfilled the eligibility criteria were included at the time of the replication study.

### Eligibility criteria for binary genome wide association analyses

All cats from the discovery stage were reviewed in order to be classified as CKD/non-CKD at entry to the study (Supplementary Methods). For the binary outcome HTN versus NT, longitudinal clinical records were reviewed with a diagnosis of HTN based on SBP > 170 mmHg, presence of ocular target organ damage and requirement for prescription of anti-hypertensive medication (Supplementary Methods).

### Genotyping and quality control

Genotyping was performed using the Illumina Infinium iSelect DNA array (Illumina, Abington, Cambridge, UK) which genotypes 62,897 SNPs across the feline genome (Bart’s and the London Genome Centre, UK). Array marker locations were adjusted to the feline genome assembly Felis catus 6.2/felcat5^46^. Quality control (QC) of the genetic data was conducted (Supplementary Methods). Eight hundred and ninety-two cats were selected for evaluation on the feline Illumina Infinium array. Sample QC was performed (Supplementary Methods) including call rate, heterozygosity, population stratification, gender and relatedness. In total 50 cats were excluded from all sample QC checks, leaving 842 cats post-QC for inclusion within the discovery GWAS.

### Phenotypes and model selection

For the GWAS analysis, an initial non-genetic statistical assessment of the phenotypic traits and potential covariates was performed, using the cats in the discovery stage, in order to select the appropriate statistical models for subsequent use. The distributions of the quantitative phenotype variables creatinine and SBP were examined visually using histogram plots and Quantile–Quantile (QQ) plots to check for normality. This resulted in the requirement to log transform creatinine (LogCreat) but not SBP. Based on prior epidemiological studies and knowledge of likely biological associations, potential covariates breed, age, sex, weight, plasma potassium, blood pressure were tested independently for association with the quantitative phenotype creatinine and the binary outcome CKD. Insufficient data were available for urine protein to creatinine ratio to be evaluated as a covariate. Similarly, the potential covariates breed, age, sex, weight, potassium and LogCreat were tested independently for association with the quantitative phenotype SBP and the binary outcome HTN. Univariate linear or logistic regression models were used for the quantitative traits or binary outcomes, respectively, to evaluate these associations. Any variables with a significant association (*P* < 0.05) were evaluated jointly in a multivariate model, and those remaining significant were selected as covariates for the GWAS. Bivariate plots and correlation statistics showing the relationship between each phenotype and covariate were also reviewed to confirm the chosen covariates. The addition of the genetic principle components 1 (PC1) and 2 (PC2) to both models was explored. Given that neither PC1 nor PC2 were significantly associated with the phenotypes of interest (SBP and creatinine), nor increased the variance explained by the multivariable model (adjusted R^2^), nor reflected any underlying relationship by breed, they were not included as covariates in the final GWAS models, in contrast to the usual practice in human GWAS. The final statistical models used age, potassium and LogCreat as covariates for SBP and HTN analyses, whereas age was the only covariate used for the analyses of creatinine and CKD.

### Genome wide association study discovery stage

All genetic analyses were performed in PLINK (v1.07). Each quantitative trait GWAS corresponded to a linear regression analysis testing each SNP, one at a time, for association with the phenotype with adjustment for covariates. Data for the 842 cats post-QC were checked for missing covariate data resulting in N = 839 cats remaining in the analysis for LogCreat and N = 817 cats for SBP (Supplementary Methods).

Logistic regression analyses were performed for the GWAS of the secondary binary outcomes HTN versus NT and CKD versus non-CKD. After removal of cats which could not be defined, 827 cats were evaluated in the binary CKD (N = 324) versus non-CKD (N = 503) whilst for HTN versus NT, 808 cats were analysed of which 172 cases were considered HTN and 636 cats NT.

Post-analysis QC checks were performed to confirm the results for each of the analyses, which resulted in filtering the results to 49,945 SNPs with MAF ≥ 5% (Supplementary Methods).

In order to select an appropriate experimental array-wide significance threshold for this new feline genotyping array, we considered all SNPs on the chip and used linkage disequilibrium (LD) pruning, according to an r^2^ threshold of 0.2 to determine the number of independent SNPs (~ 25,000) and hence the number of independent tests to adjust for in the Bonferroni multiple testing correction of 0.05/25,000. This resulted in an experimental array-wide significance threshold of *P* < 2 × 10^–6^ for all GWAS analyses.

### Replication and meta-analysis for quantitative traits SBP and creatinine

Replication analyses were performed for the quantitative traits SBP and LogCreat. As no SNPs reached experimental wide significance in the discovery stage, SNPs for inclusion in the replication stage for both LogCreat and SBP were selected on the basis of MAF > 5% and *P* < 1 × 10^–4^. For any potential follow-up SNP, a visual review of regional locus plots was performed to check for locus-level support by seeing if any SNPs in LD with the top SNP also showed evidence of association. No plots showed any major QC warnings for any of the follow-up SNPs, although due to sparse coverage, there are very few SNPs in LD. Having checked pairwise LD of the SNPs with *P* < 1 × 10^–4^, one SNP for creatinine was excluded, hence only following up pairwise LD independent SNPs at *P* < 1 × 10^–4^. This resulted in 5 follow-up SNPs for SBP and 10 for LogCreat to be taken forward for genotyping in the replication cohort.

One hundred and eighty-seven cats were selected for inclusion in the replication stage and combined meta-analysis. Genotyping and QC was performed including MAF and call rate for SNPs (Supplementary Methods) with sample QC including call rate and evaluation of covariate data (Supplementary Methods). Ultimately 14 SNPs were analysed: 5 for SBP and 9 for LogCreat. After sample QC 180 cats were available for creatinine replication analysis and 178 for SBP.

Linear genetic association analyses evaluating the replication data were performed in PLINK for LogCreat and SBP using the same covariates as the discovery stage GWAS. A meta-analysis was performed for each of the two quantitative traits using METAL software (version release 2011–03-25; http://www.sph.umich.edu/csg/abecasis/metal/)^89^. Heterogeneity between the discovery vs replication stages was checked within the meta-analysis.

As significance definitions, we declared a SNP as “formally replicated” if it reached Bonferroni corrected significance, *P* = 0.01 (for testing 5 SNPs) for SBP and *P* = 0.0056 (for testing 9 SNPs) for LogCreat, in the replication data alone, together with having concordant direction of effect between discovery and replication stages. Any SNPs reaching experimental wide significance (*P* < 2 × 10^–6^) in the combined meta-analysis of both discovery and replication data-sets together with a concordant direction of effect between discovery and replication stages are also reported as overall significant associations.

### Genetic risk score analyses

Genetic risk score (GRS) analyses were performed, combining data from multiple genetic variants: firstly constructing GRSs of the top SNPs from the discovery GWAS for each of SBP and LogCreat, in an attempt to validate the genetic contribution of these SNPs to both SBP and HTN and to LogCreat & CKD; secondly to construct GRSs based on known human CKD genes.

#### GRS of top SNPs

For BP analyses the SBP-GRS was constructed using the set of 5 follow-up SNPs reaching *P* < 1 × 10^–4^ for association with SBP. For each cat, the number of trait-increasing alleles were summed across all SNPs, and weighted according to the SNP’s beta regression effect estimate of the discovery GWAS analysis for SBP, and then averaged across all 5 SNPs, to derive the mean weighted risk score. Due to using genotyped data, the mean GRS, averaging across the number of non-missing SNPs per cat, is preferable, in order to account for any small amount of missing SNP data. The GRS was constructed using the “score” risk profile function in PLINK v1.07. Similarly, the weighted mean creat-GRS was constructed, using the 10 LD-pruned independent follow-up SNPs reaching *P* < 1 × 10^–4^ from the LogCreat analysis. The SBP-GRS was analysed for association with SBP and HTN using linear and logistic regression analyses, respectively, adjusting for age, potassium and LogCreat as covariates. The creat-GRS was analysed for association with LogCreat and CKD using linear and logistic regression analyses, respectively, adjusting for age as the only covariate, as in the primary analyses. Initially, the GRS were constructed and tested within the same discovery sample of cats (N = 827 LogCreat and CKD, N = 808 SBP and HTN). Then as the main analysis, the SBP-GRS and creat-GRS (with only 9 of the 10 LogCreat SNPs available in the replication data post-QC) were constructed and tested using the independent replication sample of cats (N = 180).

#### GRS of human CKD genes

For the purposes of this exploratory analysis, we only considered LogCreat and not SBP, knowing from our primary GWAS analyses, that LogCreat analysis had shown more significant results than the SBP analysis. At the time of analysis, the most recent publication was used to identify loci associated with renal function^38^. The gene names associated with these loci (n = 53) were searched using Ensembl 90 and UCSC databases (Sep.2011 (ICGSC Felis_catus 6.2/felCat5) for predicted comparable feline genes with concurrent mapping of known SNP locations from the feline array^78^. Predicted feline genetic loci were identified for 46 human loci and the closest SNP on the feline array identified. Where multiple SNPs fell within the predicted gene location, the SNP with the highest significance value was selected. Gene and SNP locations were checked against the latest Felis_catus_9.0 genome assembly (NCBI genome data viewer (Felis_catus_9.0 (GCF000181335.3))^85^. As these 46 SNPs were not genotyped for follow-up in the replication sample, this GRS analysis could only be performed within the discovery dataset. Therefore, in order to have independent subsets of the data for model-building vs analysis-testing, we randomly split the N = 839 discovery cats from the LogCreat analysis with non-missing LogCreat and age variables for phenotype and covariate data according to a 70:30 training: testing ratio, with N = 587 cats in the training subset and N = 252 cats in the testing subset. A new training association analysis was run to test these 46 CKD SNPs for association with LogCreat, adjusted for age, restricted only to the 587 cats in the training dataset. One of the 46 closest CKD SNPs was not available for this analysis, hence a total of 45 SNPs remained. Similarly, to the first GRS analysis construction above, the “score” function was used in PLINK to construct the GRS of these 45 SNPs, on the 252 cats in the testing dataset, weighted according to the beta effect estimates from the training analysis results, aligned to the equivalent effect alleles, giving the average score per non-missing SNP, for the mean weighted GRS. In addition to the main linear regression analysis analysing the 46-SNP human-CKD GRS as a continuous score variable for association with LogCreat, adjusted for age, for all 252 cats in the testing dataset, a quintile analysis was also performed to evaluate if there was any significant difference in creatinine levels comparing the cats in the top 20% quintile of the human-CKD-gene genetic risk score vs the cats in the lowest 20% risk group.

### Gene-set enrichment and pathway-based analysis

Gene-set enrichment and pathway-based analysis (GSEA) was performed^90^. A nominal *p* < *0.01* was used to filter SNPs from the GWAS analysis for GSEA. Ensembl FelCat 5 gene annotations (https://hgdownload.soe.ucsc.edu/goldenPath/felCat5/database/) were used to assign SNPs to genes if they were either within the genomic sequence of the gene, or within the 5 Kb upstream/downstream flanking regions in order to include SNPs within regulatory regions^91^. The Kyoto Encyclopedia of Genes and Genomes pathway (KEGG), Gene Ontology (GO) biological process, and msigdb Hallmark databases were used for functional annotation and enrichment analyses^92–94^. To avoid testing narrow or broad categories, only categories with more than 10 and less than 500 genes were included. A Fisher’s exact test was performed to test for over-representation of the significant genes in each gene-set with BH (Benjamini & Hochberg) correction for multiple testing.

## Supplementary Information

Supplementary Information.

## Data Availability

The datasets generated during and/or analysed during the current study are available in the Royal Veterinary College repository, https://rvc-repository.worktribe.com/output/1443487.
