# Supplementary material for: First genome-wide association study investigating blood pressure and renal traits in domestic cats
Source: Sci Rep. 2022 Feb 3;12:1899. doi: 10.1038/s41598-022-05494-3 (PMC8813908; doi:10.1038/s41598-022-05494-3)
Supplement: Supplementary file 1 — Supplementary Information. [file 41598_2022_5494_MOESM1_ESM.docx]

**Supplemental Material**

**First Genome-Wide Association Study Investigating Blood Pressure and Renal Traits in Domestic Cats**

Authors: R.E. Jepson*^1,+^, H. Warren^3,+^, M.D. Wallace^1,4^, H.M. Syme^1^, J. Elliott^2^, P.B. Munroe^3^

^1^Department of Clinical Services and Sciences, Royal Veterinary College, London, UK

^2^Department of Comparative Biomedical Sciences, Royal Veterinary College, London, UK

^3^Department of Clinical Pharmacology, Barts and the London School of Medicine and Dentistry, Queen Mary University of London, London, UK

^4^Nuffield Department of Medicine, Wellcome Centre for Human Genetics, University of Oxford, Oxford, UK

**Supplementary Methods**

**Feline samples and clinical phenotyping**

The geriatric feline clinic at the Royal Veterinary College recruits healthy cats ≥ 9 years or those with either CKD or HTN. As part of the longitudinal health monitoring, healthy cats were routinely examined every 6 months, whilst cats with CKD and HTN were examined approximately every 6-8 weeks and were provided with standardised care free of charge in accordance with current International Renal Interest Society (IRIS) recommendations.^a^ Healthy cats were routinely sampled for evaluation of renal function (serum biochemical profile and urinalysis where possible) and SBP assessed using the Doppler technique on each visit, whilst cats with either HTN and/or CKD were sampled at every other visit (12-16 weeks) and SBP assessed at every visit. This longitudinal monitoring programme has been recruiting cats since 1998 with available searchable computerised records since that time.

Genomic DNA was extracted from whole blood cell pellets using a commercially available extraction kit (GenElute Blood Genomic DNA Kit, Sigma-Aldrich/Merck KGaA). For inclusion in the study, assessment of total thyroxine concentration < 40 nmol/l either at the sample date of interest, up to 3 months prior or 12 months post-sample date was required in order to confirm a euthyroid status.

^a^www.iris-kidney.com

**Case exclusion criteria:**

Cats were excluded where the presence of concurrent medical conditions (e.g. diabetes mellitus, neoplasia). Cats with a diagnosis of hyperthyroidism and those receiving medical anti-thyroid medication were excluded from the study. Cats that had undergone previous surgical thyroidectomy were included in the study but only on the basis that total thyroxine at the time of enrolment confirmed euthyroid status. There were 8 cats in both the creatinine and SBP analyses that had undergone surgical thyroidectomy but were euthyroid at the time of inclusion in both analyses (Total thyroxine (N=8) median 18.4 range 4.0-39.0 nmol/l), hence these cases remained in the GWAS analyses. Information relating to prescribed medications at the time of enrolment was evaluated. Cats were excluded from the GWAS assessing renal function if they were receiving medications that could influence renal function (e.g. angiotensin converting enzyme inhibitors (ACEi), angiotensin receptor blockers (ARB)) or if there was strong indication for a pre-renal component to the azotaemia (e.g. cat admitted at same hospitalised visit for intravenous fluid therapy). Cats were excluded from the GWAS evaluation of SBP if they were receiving medications that could influence BP (e.g. ACEi, ARB, calcium channel blockers, beta-blockers). Cats with known concurrent disease such as neoplasia, diabetes mellitus and congestive heart failure were also excluded.

**Definition of chronic kidney disease (CKD) for binary GWAS analysis**

A diagnosis of azotemic CKD at the time of entry to the study was made on the basis of a compatible clinical history and physical examination findings in addition to a single creatinine concentration >177 µmol/l together with a urine specific gravity <1.035 or at least two creatinine concentrations >177 µmol/l a minimum of 4 weeks apart. Cats that were non-azotemic (creatinine <177 µmol/l) and/or those with adequate urine concentrating ability (USG >1.035) were considered non-CKD. Cat had to be followed for a minimum of 12 months to be classified as non-azotaemic. Cats were excluded if they were only seen at one visit and/or there was insufficient information to determine a diagnosis of CKD.

**Definition of hypertension (HTN) for binary GWAS analysis**

A diagnosis of systemic HTN was made on the basis of SBP >170 mmHg on a single occasion if in association with ocular target organ damage (TOD; e.g. hypertensive retinopathy/choroidopathy, gross hyphema, retinal vessel tortuosity, retinal haemorrhage) or SBP > 170 mmHg on at least 2 occasions where no ocular TOD was present. Cats which were already receiving anti-hypertensive medication (amlodipine besylate) on their initial visit, and which had been excluded from the quantitative analysis of SBP, were included in the binary analysis as HTN cats based on prior diagnosis and instigation of anti-hypertensive treatment. Cats where only a single visit was available, and it was not possible to determine HTN/NT status, were excluded from the analysis.

**Genotyping and quality control**

Genotyping was performed using the Illumina Infinium iSelect DNA array (Illumina, Abington, Cambridge, UK) which genotypes 62,897 SNPs across the feline genome (Bart’s and the London Genome Centre, UK). Array marker locations were adjusted to the feline genome assembly Felis catus 6.2/felcat5.^46^

Quality control (QC) of the genetic data was conducted, adapting equivalent practices from standard approaches used in human GWAS (Figure 1a). SNP QC was performed using PLINK (v1.07) including removal of insertion/deletion polymorphisms (n=7) and non-genotyped SNPs (n=63) and filtering according to genotype call rate <90%, Hardy Weinberg equilibrium (HWE) threshold of *P*< 1×10^-6^ and minor allele frequency (MAF) of < 1%, with 57,674 SNPs remaining for all analyses.

Eight hundred and ninety-two cats were selected for evaluation on the feline Illumina Infinium array (Figure 1a). For sample QC, call rate, heterozygosity, population stratification, gender and relatedness were assessed. Thresholds for a sample call rate of < 97% and heterozygosity rates of > 4SD from the mean were applied and resulted in exclusion of 5 and 8 cats respectively. In human GWAS, Principal Component Analysis (PCA) is performed to check that all samples are of homogeneous ancestry to avoid population confounding, as the principal components (PCs) of the genetic data are accurate representations of ancestral variation between individuals. We were interested to investigate whether the PCs from the feline GWAS reveal any population structure of cats, for example according to breed. We therefore used PLINK to perform PCA and extract the first two PCs PC1 and PC2 from the post-QC genetic data. Plots of PC1 vs PC2 were produced for all cats, with colour-coding to compare DSH and DLH cat groups. This revealed a large degree of overlap between DSH and DLH cats, with no distinct clustering by breed (Supplemental Fig S1), indicating that these cats did not represent separate populations genetically and therefore could be combined for further data analysis without requirement for stratification. Evaluating the PCA plots further, two cats were excluded on the basis of being outliers (> 3SD from the mean) for both PC1 and PC2. Hence 15 cats were excluded according to call-rate, heterogeneity or PCs.

A gender check identified 14 cats with apparent gender discordance between the genetically-inferred sex and phenotypic sex (Figure 1a). The relatedness of cats was assessed using identity by descent (IBD) estimates calculated in PLINK and cats with PiHat >0.5 (indicating 1^st^ degree relatedness of parent-offspring or sibling relationship) were identified. Twenty-three pairs (45 cats, as 1 cat in 2 pairs) with PiHat > 0.5 were identified. The results of the gender check and relatedness were then combined together. In order to restrict the sample to a set of unrelated cats to remove any population structure bias, one member of each related pair must be excluded prior to GWAS analysis. If either cat corresponded to a sample failing the gender check, this cat was excluded; otherwise the cat with the lowest call rate was excluded.

Of the 23 related pairs, two pairs included a cat with gender failure, hence two cats were removed due to both gender discordance and relatedness. For the remaining 21 related pairs, 21 cats were removed according to the minimum call rate. Finally, the remaining 12 cats from the 14 samples with gender discordance were removed. Hence 35 cats were excluded according to gender discordance or relatedness.

Therefore, in total 50 cats were excluded from all sample QC checks, leaving 842 cats post-QC for inclusion within the discovery GWAS (Figure 1a).

**Evaluation of post-QC samples for missing covariate data for discovery GWAS**

Data for the 842 cats post-QC were checked in case of any missing covariate data. For LogCreat analysis, 839 cats were included in the GWAS with only N=3 cats missing for age. For SBP, 817 cats were included with 15 excluded due to anti-hypertensive therapy (amlodipine besylate) and 10 excluded due to missing covariates (N=3 age and N=7 potassium).

**Genotyping for replication stage**

Genomic DNA was extracted as previously and a Fluidigm genotyping system (Bart’s and the London Genome Centre, UK) used for genotyping selected SNPs across the replication cohort. Control samples of each genotype for each SNP from the discovery stage were selected and included to ensure comparability of genotyping techniques.

**Quality Control for Replication Data and Samples**

SNPs were checked to have similar MAF between discovery and replication data: only 1 SNP (chrB1_225124311) showed different frequencies and was still analysed but was flagged (and did not end up reaching significant levels for reporting). One SNP from the creatinine analysis was removed (chrB3_157576167) due to a genotype call rate of 21% and one SNP (chrB1_176960684) was flagged due to a call rate of 62%. All remaining SNPs had call rates >0.9 and therefore 14 SNPs were analysed: 5 for SBP and 9 for creatinine. After performing sample QC, 3 cats were excluded due to low call rate <0.9 and 4 for missing covariate data, leaving 180 cats available for the creatinine replication analysis (Figure 1b). A further two cats were excluded due to missing covariate data for the SBP analysis leaving 178 cats for the SBP replication analysis. Ultimately, 13 SNPs were taken forwards to metanalysis; 9 for LogCreat and 4 for SBP.

**Gene mapping for SNP reaching experimental wide significance in quantitative creatinine GWAS**

Using Felis_catus v9.0, the SNP which reached significance, chrD1.10258177, in the meta-analysis associated with creatinine relocates to position ChrD1:8507247.^b^ NCBI genome data viewer was used to identify predicted genes within 1 megabase pairs (Mbp) using the Felis catus 9.0/Felcat 9 (GCF_ 000181335.3) genome assembly. The gene list produced was then compared to genes in closest proximity to known renal loci from human medicine from the study by Wuttke and colleagues.^c^ Genes identified within 1 Mbp were also reviewed using NCBI gene for any potential pathophysiological relevance with the trait of interest and GWAS catalog^c^ for any known renal associated GWAS.

*^b^Samaha, G. et al. Mapping the genetic basis of diabetes mellitus in the Australian Burmese cat (Felis catus). Scientific Reports.* ***10****, 1-12 (2020)*

*^c^ Wuttke, M. et al. A catalog of genetic loci associated with kidney function from analyses of a million individuals. Nat. Genet.* ***51****, 957–972 (2019)*

*^c^https://www.ebi.ac.uk/gwas/*

**Figure S1: Principal component analysis comparing domestic shorthair and domestic longhair cats**


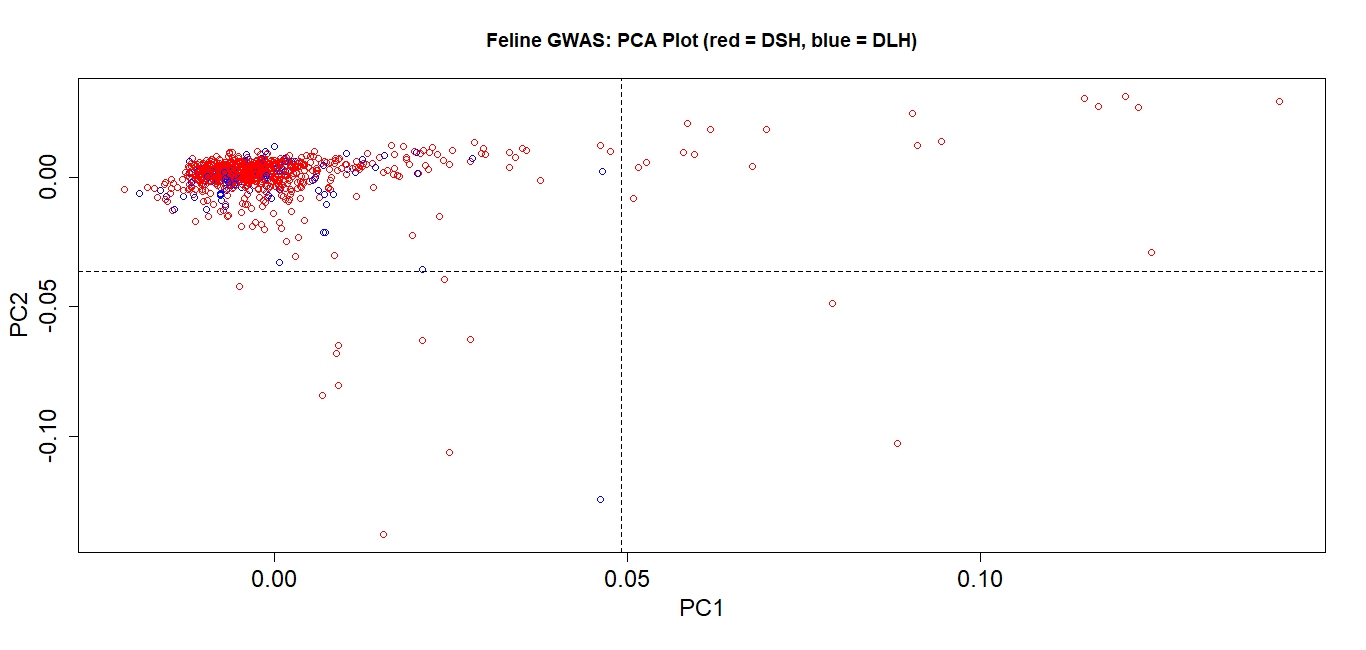


*Principal component analysis plot demonstrating overlap between domestic shorthair (DSH, red) and domestic longhair (DLH, blue) cats. Cats falling outside principal components 1 and 2 (PC1 and PC2) (>3SD from the mean) were excluded (N=2).*

**Figure S2: Quantile-quantile plot for GWAS evaluating Log creatinine as a quantitative trait in the discovery stage**

**
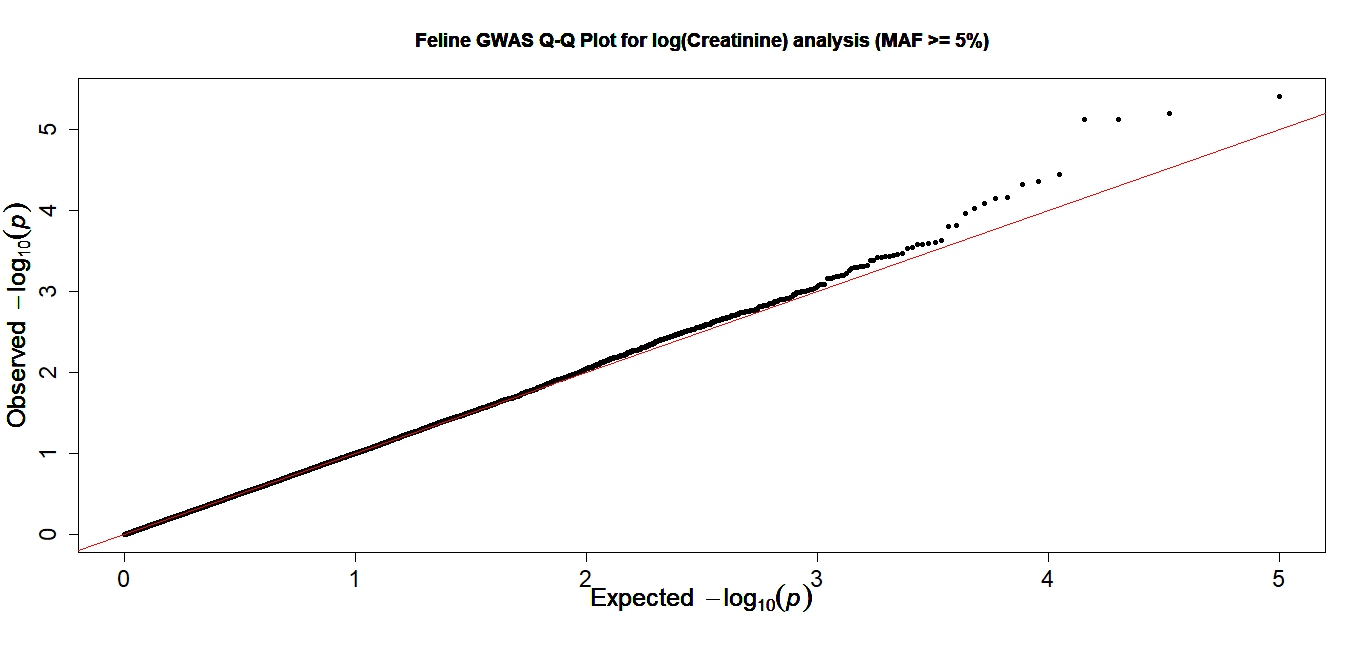
**

*QQ plot of results for Log Creatinine from genome wide association study discovery stage (N=839). The black curves are based on all single nucleotide polymorphisms with minor allele frequency ≥5% (Lamda 1.009). The P values have been derived from a linear regression analysis.*

**Figure S3: Quantile-Quantile plot for GWAS evaluating systolic blood pressure as a quantitative trait in the discovery stage**

**
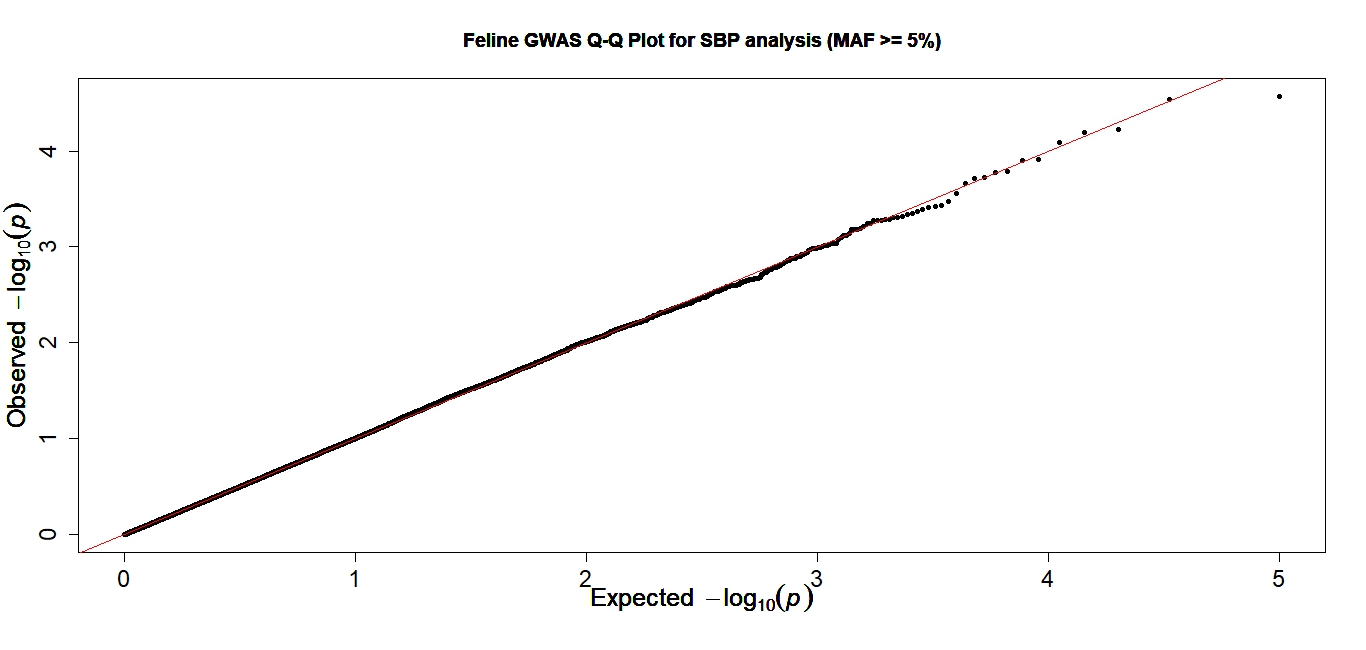
**

*QQ plot of results for systolic blood pressure from genome-wide association study discovery stage (N=817). The black curves are based on all single nucleotide polymorphisms with minor allele frequency ≥5% (Lamda 1.005). The P values have been derived from a linear regression analysis.*

**Figure S4: Manhattan plot for binary case-control GWAS analysis evaluating cats with and without a diagnosis of chronic kidney disease in the discovery stage**

**
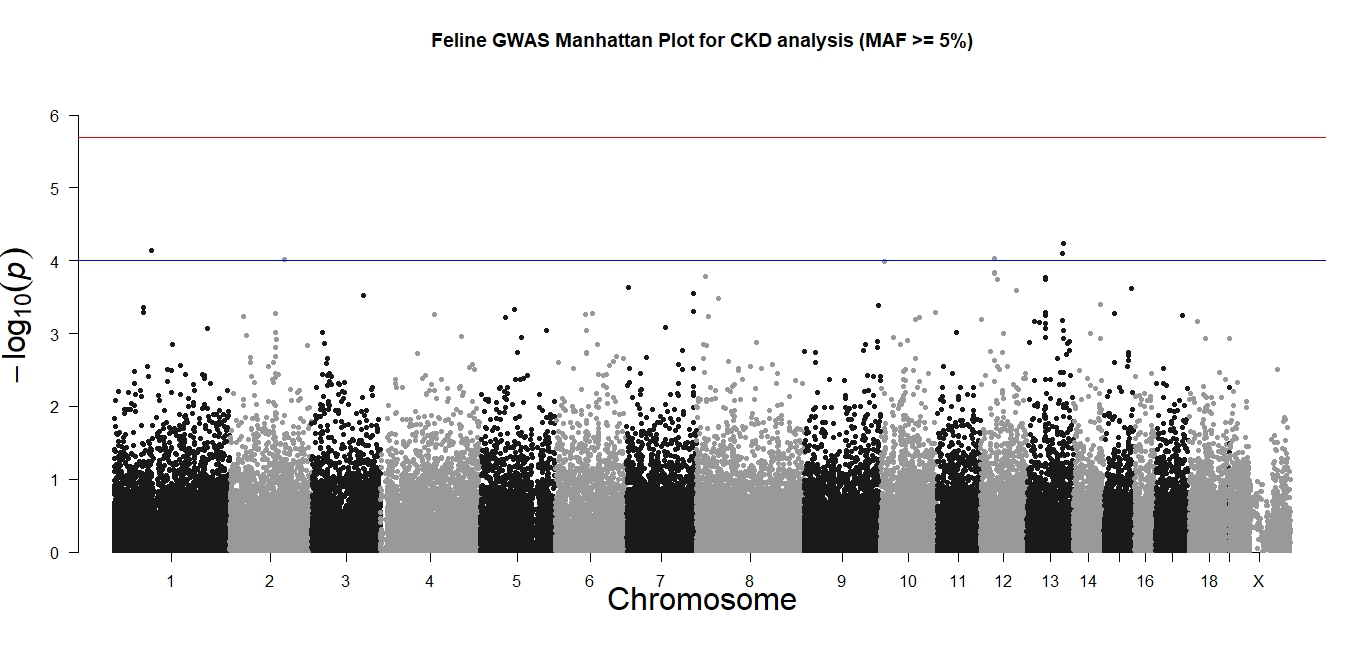
**

*Manhattan plot of the discovery genome-wide association study in 827 cats (N=324 chronic kidney disease and N=503 non-chronic kidney disease). The y axis shows the -log10 P values for single nucleotide polymorphisms (SNPs) with minor allele frequency ≥5% and the x axis shows their chromosomal positions. Horizontal blue and red lines represent the thresholds of P =1 x 10^-4^ and P = 2×10^-6^. No SNPs reached experimental-wide significance (P < 2×10^-6^) at the discovery stage.*

**Figure S5: Manhattan plot for binary case-control GWAS analysis evaluating hypertensive and normotensive cats in the discovery stage**

**
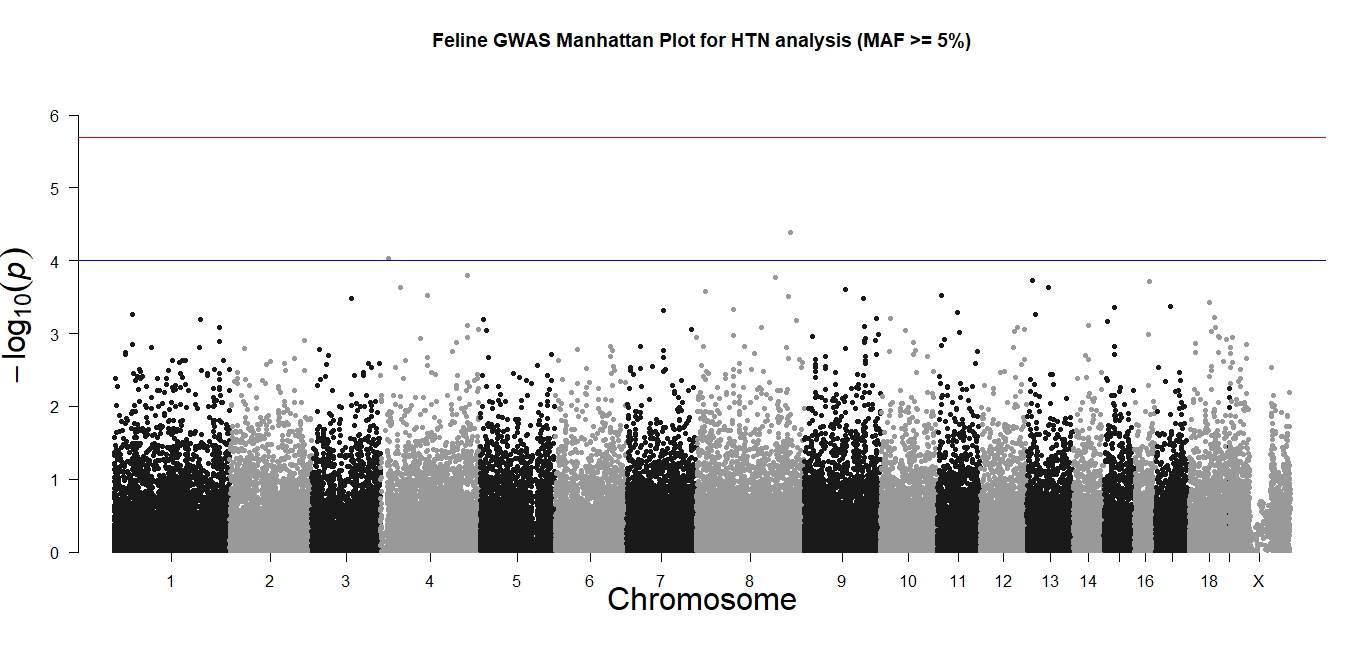
**

*Manhattan plot of the discovery genome-wide association study in 808 cats (N=172 hypertensive and N=636 normotensive). The y axis shows the -log10 P values for single nucleotide polymorphisms (SNPs) with minor allele frequency ≥5% and the x axis shows their chromosomal positions. Horizontal blue and red lines represent the thresholds of P =1 x 10^-4^ and P = 2×10^-6^, respectively. No SNPs reached experimental-wide significance (P < 2×10^-6^) at the discovery stage.*

**Figure S6: Bivariate plots comparing GWAS discovery results of the quantitative LogCreatinine trait and binary outcome of chronic kidney disease**

**
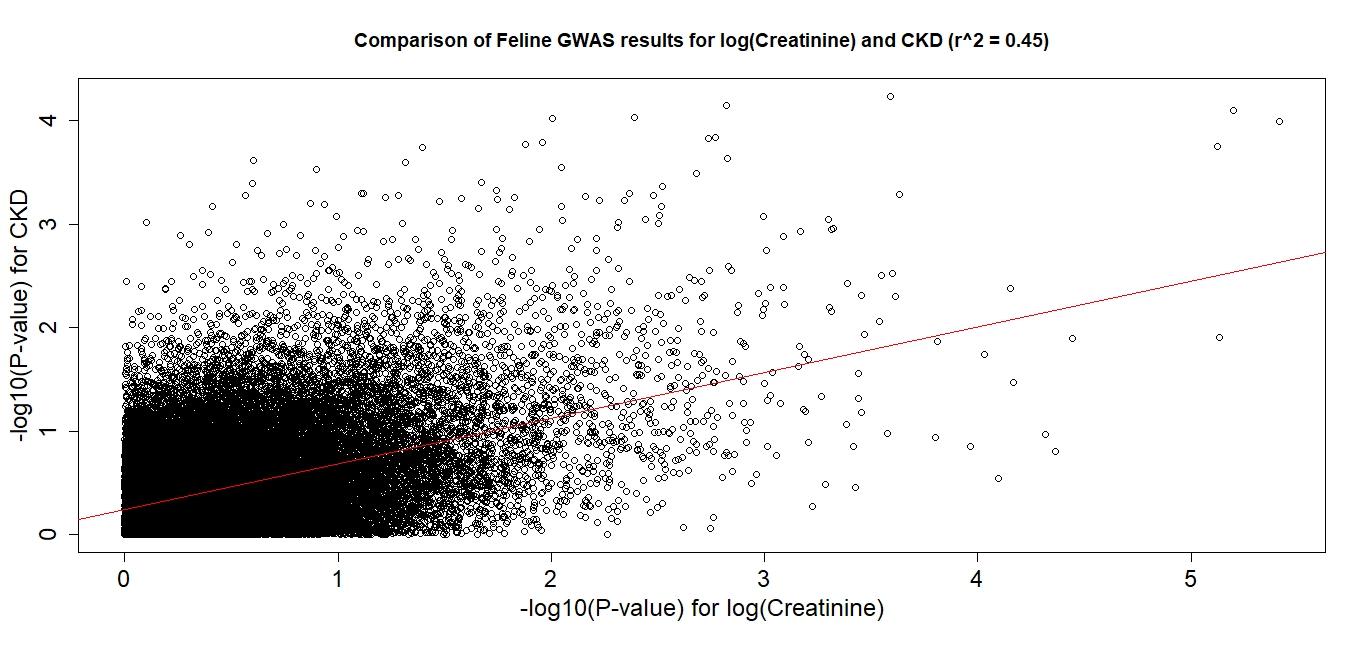
**

*The –log10(P-value) association results from the discovery genome-wide association analyses are plotted for all single nucleotide polymorphisms (SNPs) with minor allele frequency ≥5% comparing the results for log(Creatinine) on the x-axis with the results for chronic kidney disease (CKD) on the y-axis. The red fitted line from the linear regression illustrates the linear relationship with a positive correlation r^2^=0.45; P<2×10^-16^.*

**Figure S7: Bivariate plots comparing GWAS discovery results of the quantitative systolic blood pressure trait and binary outcome of Hypertension**

**
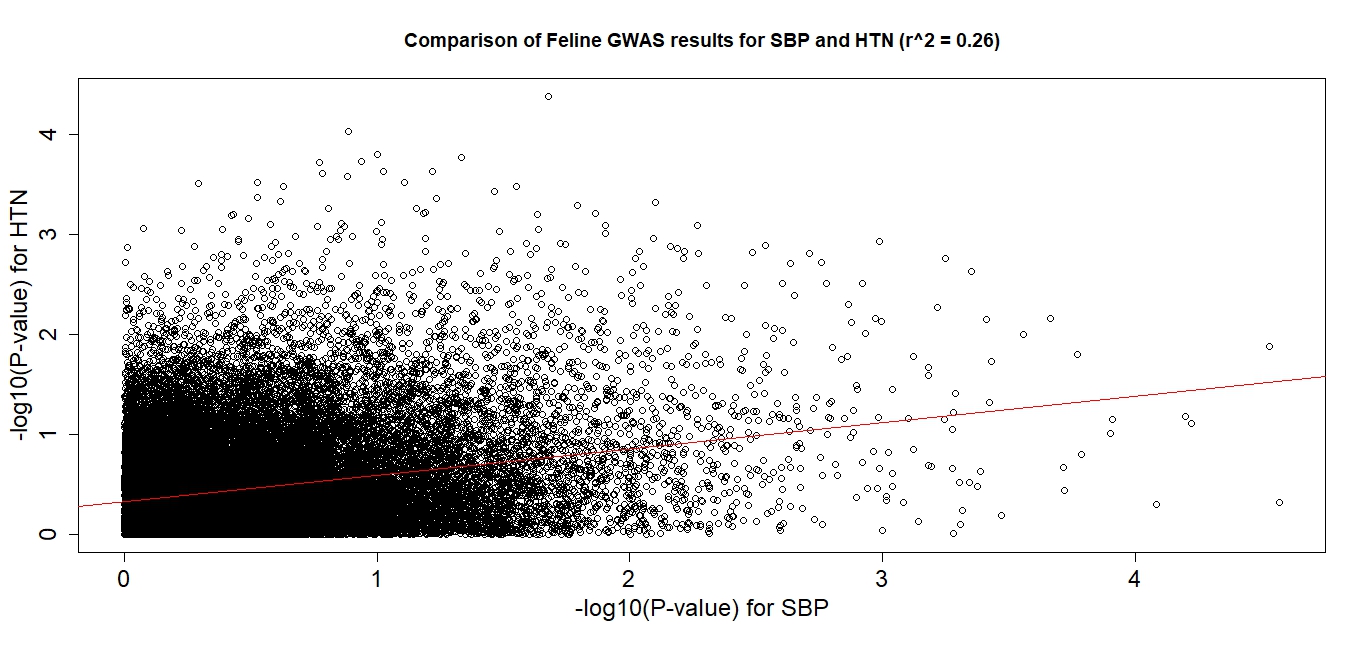
**

*The –log10(P-value) association results from the discovery genome-wide association analyses are plotted for all single nucleotide polymorphisms (SNPs) with minor allele frequency ≥5% comparing the results for systolic blood pressure (SBP) on the x-axis with the results for hypertension (HTN) on the y-axis. The red fitted line from the linear regression illustrates the linear relationship with a positive correlation r^2^=0.26; P<2×10^-16^.*

**Table S1: Clinicopathological data for cats defined as chronic kidney disease and non-chronic kidney disease at baseline**

|  | **CKD** | | **Non-CKD** | | **p** |
| --- | --- | --- | --- | --- | --- |
| **Variable** | **Median**  **(25^th^, 75^th^ percentile)** | **Number (n)** | **Median**  **(25^th^, 75^th^ percentile)** | **Number (n)** |  |
| **Age (years)** | 14.8 (12.0, 16.6) | 324 | 13.0 (11.0, 15.0) | 503 | <0.0001 |
| **Sex (by computerised database)** | Female entire  Female neutered  Male entire  Male neutered | 2  165  4  153 | Female entire  Female neutered  Male entire  Male neutered | 5  243  2  253 | - |
| **Breed** | Domestic longhair  Domestic shorthair | 44  280 | Domestic longhair  Domestic shorthair | 60  443 | - |
| **Body weight (kg)** | 3.78 (3.20, 4.56) | 313 | 4.00 (3.34, 4.88) | 492 | 0.006 |
| **Creatinine (µmol/l)** | 218.4 (193.8, 251.7) | 324 | 134.0 (116.7, 154.0) | 503 | <0.0001 |
| **Phosphorus (mmol/l)** | 1.39 (1.18, 1.72) | 322 | 1.30 (1.11, 1.48) | 503 | <0.0001 |
| **Potassium (mmol/l)** | 4.08 (3.70, 4.40) | 311 | 4.00 (3.74, 4.34) | 497 | 0.867 |
| **Packed cell volume (%)** | 34 (30, 38) | 321 | 36 (32, 40) | 495 | <0.0001 |
| **Systolic blood pressure (mmHg)** | 145.6 (130.8, 162.4) | 319 | 131.6 (117.2, 149.2) | 503 | <0.0001 |
| **Urine specific gravity** | 1.018 (1.016, 1.024) | 231 | 1.039 (1.027, 1.050) | 339 | <0.0001 |

*CKD; Cats diagnosed with chronic kidney disease (CKD) at the baseline visit (i.e. first visit where concurrent systolic blood pressure (SBP) and creatinine data available), non-CKD; cats that did not have a diagnosis of CKD at their baseline visit (i.e. first visit where concurrent SBP and creatinine data were available).*

**Table S2: Clinicopathological data for cats defined as hypertensive and normotensive**

|  | **Hypertensive** | | **Normotensive** | | **p** |
| --- | --- | --- | --- | --- | --- |
| **Variable** | **Median**  **(25^th^, 75^th^ percentile)** | **Number (n)** | **Median**  **(25^th^, 75^th^ percentile)** | **Number (n)** |  |
| **Age (years)** | 14.7 (10.7, 16.2) | 172 | 13.0 (11.0, 15.2) | 636 | <0.0001 |
| **Sex (by computerised database)** | Female entire  Female neutered  Male entire  Male neutered | 2  93  1  76 | Female entire  Female neutered  Male entire  Male neutered | 4  311  5  316 |  |
| **Breed** | Domestic longhair  Domestic shorthair | 20  152 | Domestic longhair  Domestic shorthair | 84  552 |  |
| **Body weight (kg)** | 3.86 (2.78, 4.65) | 163 | 3.98 (3.29, 4.80) | 622 | 0.412 |
| **Creatinine (µmol/l)** | 182.8 (151.3, 233.6) | 172 | 147.8 (123.0, 183.5) | 636 | <0.0001 |
| **Phosphorus (mmol/l)** | 1.37 (1.18, 1.60) | 171 | 1.30 (1.12, 1.54) | 636 | 0.035 |
| **Potassium (mmol/l)** | 3.90 (3.60, 4.29) | 172 | 4.10 (3.80, 4.40) | 636 | 0.001 |
| **Packed cell volume (%)** | 35 (32, 38) | 171 | 35 (32, 40) | 636 | 0.991 |
| **Systolic blood pressure (mmHg)** | 165.2 (145.6, 195.8) | 172 | 131.0 (117.6, 143.6) | 636 | <0.0001 |
| **Systolic blood pressure at visit where diagnosed with hypertension (mmHg)** | 186.2 (176.9, 206.8) | 172 |  |  | <0.0001 |
| **Urine specific gravity** | 1.022 (1.016, 1.033) | 126 | 1.033 (1.021, 1.048) | 426 | <0.0001 |

*Hypertensive; Longitudinal data was evaluated for all cats such that cats that had ever been diagnosed with hypertension could be identified. Clinical data for cats at the visit where hypertension was diagnosed is presented, Normotensive; Cats which were never diagnosed with systemic hypertension, baseline visit clinical data is presented (i.e. first visit where combined systolic blood pressure and creatinine data is available).*

**Table S3: Binary genome wide analysis of chronic kidney disease versus non-chronic kidney disease cats and hypertensive and normotensive cats in the discovery stage**

| **Discovery** | | | | | | | | | |
| --- | --- | --- | --- | --- | --- | --- | --- | --- | --- |
| **SNP** | **CHR** | **BP** | **A1** | **A2** | **n** | **Freq1_disc** | **OR_disc** | **SE_disc** | **P_disc** |
| **CKD vs nonCKD** | | | | | | | | | |
| chrD4.73841594 | 13 | 74643509 | A | T | 827 | 0.22 | 1.65 | 0.12 | 5.82 x10^-5^ |
| chrA1.92085844 | 1 | 76692448 | G | C | 820 | 0.2946 | 1.56 | 0.11 | 7.17 x10^-5^ |
| chrD4.72377931 | 13 | 73436741 | A | T | 827 | 0.1188 | 1.84 | 0.16 | 8.06 x10^-5^ |
| chrUn26.5427867 | 12 | 28580565 | G | C | 826 | 0.06599 | 2.27 | 0.21 | 9.32 x10^-5^ |
| chrA2.174204010 | 2 | 112863084 | A | T | 827 | 0.3717 | 0.65 | 0.11 | 9.47 x10^-5^ |
| **NT vs HTN** | | | | | | | | | |
| chrC1.214195187 | 8 | 193060590 | A | T | 807 | 0.12 | 2.12 | 0.18 | 4.15 x10^-5^ |
| chrB1.19817607 | 4 | 16530091 | G | C | 808 | 0.20 | 1.81 | 0.15 | 9.32 x10^-5^ |

*SNP; single nucleotide polymorphism (Named from Illumina Feline Infinium Array), CHR; chromosome (FelCat5; Felis_catus-6.2 Genome assembly), BP; base pairs (FelCat5; Felis_catus-6.2 Genome assembly), A1; minor allele, A2; major allele, n; number, disc; discovery cohort, freq1; frequency of minor allele, OR; Odds ratio, SE; standard error, p; significance.*

**Table S4: Single-SNP association results from the 70% discovery training subset of N=587 cats for the 45 feline SNPs corresponding to the known human CKD genes**

| **Human CKD Gene** | **Closest Feline SNP** | **Effect Allele** | **N (Full Discovery)** | **BETA (Full Discovery)** | **SE (Full Discovery)** | **P-value (Full Discovery)** | **N (Training Subset)** | **BETA (Training Subset)** | **SE (Training Subset)** | **P-value (Training Subset)** |
| --- | --- | --- | --- | --- | --- | --- | --- | --- | --- | --- |
| SLC34A1 | chrA1.211817204 | A | 834 | 0.010 | 0.019 | 0.608 | 583 | -0.004 | 0.024 | 0.879 |
| DAB2 | chrA1.254030510 | A | 839 | -0.016 | 0.019 | 0.409 | 587 | -0.003 | 0.023 | 0.912 |
| KBTBD2 | chrA2.188139493 | A | 839 | 0.031 | 0.018 | 0.079 | 587 | 0.036 | 0.022 | 0.101 |
| RNF32 | chrA2.215892075 | A | 839 | -0.019 | 0.028 | 0.509 | 587 | -0.017 | 0.034 | 0.630 |
| WNT7A | chrA2.69341715 | A | 839 | 0.033 | 0.025 | 0.190 | 587 | 0.059 | 0.031 | 0.058 |
| ALMS1 | chrA3.116874036 | G | 839 | -0.071 | 0.028 | **0.011** | 587 | -0.079 | 0.034 | **0.020** |
| DDX1 | chrA3.142242750 | G | 839 | -0.003 | 0.017 | 0.876 | 587 | -0.018 | 0.021 | 0.374 |
| TP53INP2 | chrA3.5094773 | A | 839 | -0.026 | 0.021 | 0.207 | 587 | -0.029 | 0.025 | 0.251 |
| BCAS1 | chrA3.9046761 | G | 838 | 0.009 | 0.017 | 0.587 | 586 | 0.006 | 0.021 | 0.756 |
| NFKB1 | chrB1.147269226 | A | 839 | 0.012 | 0.018 | 0.483 | 587 | 0.027 | 0.022 | 0.223 |
| STC1 | chrB1.41912275 | A | 839 | -0.011 | 0.019 | 0.574 | 587 | -0.024 | 0.024 | 0.326 |
| SLC22A2 | chrB2.168604887 | G | 838 | -0.013 | 0.018 | 0.455 | 586 | -0.016 | 0.021 | 0.437 |
| VEGFA | chrB2.52741802 | A | 839 | -0.029 | 0.025 | 0.236 | 587 | -0.054 | 0.030 | 0.072 |
| GATM | chrB3.63117160 | G | 839 | 0.010 | 0.017 | 0.585 | 587 | 0.023 | 0.022 | 0.280 |
| INO80 | chrB3.72235874 | A | 839 | 0.053 | 0.026 | **0.039** | 587 | 0.078 | 0.032 | **0.014** |
| INHBC | chrB4.102280228 | C | 839 | 0.045 | 0.025 | 0.072 | 587 | 0.042 | 0.031 | 0.180 |
| WDR37 | chrB4.1407580 | G | 839 | -0.023 | 0.022 | 0.301 | 587 | -0.052 | 0.028 | 0.061 |
| SLC6A13 | chrB4.45984274 | A | 837 | 0.049 | 0.033 | 0.130 | 585 | 0.102 | 0.041 | **0.013** |
| TSPAN9 | chrB4.46976461 | A | 839 | -0.003 | 0.020 | 0.881 | 587 | -0.017 | 0.024 | 0.472 |
| PTPRO | chrB4.60508156 | A | 839 | -0.015 | 0.020 | 0.437 | 587 | -0.014 | 0.025 | 0.558 |
| DPEP1 | chrC1.10172494 | G | 839 | 0.059 | 0.025 | **0.019** | 587 | 0.076 | 0.030 | **0.011** |
| SYPL2 | chrC1.109931014 | C | 839 | 0.012 | 0.017 | 0.479 | 587 | 0.015 | 0.021 | 0.481 |
| LASS2 | chrC1.120143709 | G | 839 | 0.021 | 0.034 | 0.534 | 587 | 0.024 | 0.043 | 0.578 |
| CASP9 | chrC1.14778936 | A | 839 | -0.076 | 0.058 | 0.195 | 587 | -0.146 | 0.071 | **0.040** |
| LRP2 | chrC1.170556116 | A | 839 | 0.021 | 0.018 | 0.248 | 587 | 0.031 | 0.022 | 0.164 |
| CPS1 | chrC1.217015208 | NA | NA | NA | NA | NA | NA | NA | NA | NA |
| IGFBP5 | chrC1.224634264 | A | 838 | 0.036 | 0.020 | 0.081 | 586 | 0.047 | 0.025 | 0.062 |
| SKIL | chrC2.109454933 | A | 837 | -0.021 | 0.017 | 0.237 | 585 | -0.011 | 0.021 | 0.586 |
| TFDP2 | chrC2.131531541 | A | 839 | -0.025 | 0.017 | 0.144 | 587 | -0.018 | 0.022 | 0.414 |
| ETV5 | chrC2.93528035 | A | 827 | 0.008 | 0.019 | 0.673 | 581 | 0.026 | 0.023 | 0.273 |
| AP5B1 | chrD1.149261025 | A | 839 | -0.019 | 0.045 | 0.677 | 587 | -0.051 | 0.058 | 0.378 |
| A1CF | chrD2.361199 | A | 839 | -0.005 | 0.032 | 0.870 | 587 | -0.040 | 0.041 | 0.321 |
| PIP5K1B | chrD4.716786 | A | 839 | -0.038 | 0.018 | **0.038** | 587 | -0.035 | 0.022 | 0.115 |
| BCAS3 | chrE1.54320951 | C | 838 | 0.041 | 0.017 | **0.013** | 586 | 0.046 | 0.020 | **0.023** |
| CDK12 | chrE1.70836060 | G | 839 | -0.018 | 0.020 | 0.380 | 587 | -0.002 | 0.025 | 0.940 |
| SLC47A1 | chrE1.7968421 | G | 839 | -0.006 | 0.017 | 0.734 | 587 | -0.015 | 0.021 | 0.473 |
| SIPA1L3 | chrE2.21926537 | G | 839 | 0.007 | 0.017 | 0.695 | 587 | 0.007 | 0.021 | 0.744 |
| SLC7A9 | chrE2.27724210 | A | 837 | 0.003 | 0.018 | 0.861 | 585 | 0.002 | 0.022 | 0.922 |
| UMOD | chrE3.43872169 | A | 839 | -0.001 | 0.017 | 0.939 | 587 | -0.008 | 0.021 | 0.699 |
| CACNA1S | chrF1.65127677 | C | 809 | 0.014 | 0.018 | 0.417 | 566 | 0.005 | 0.022 | 0.817 |
| NFATC1 | chrUn1.711123 | A | 839 | -0.030 | 0.028 | 0.283 | 587 | -0.032 | 0.034 | 0.340 |
| TMEM60 | chrUn18.689942 | G | 839 | 0.019 | 0.029 | 0.511 | 587 | 0.045 | 0.038 | 0.231 |
| MPPED2 | chrUn18.8112547 | A | 839 | 0.015 | 0.020 | 0.446 | 587 | 0.015 | 0.024 | 0.534 |
| WDR72 | chrUn30.4410755 | G | 839 | -0.030 | 0.038 | 0.439 | 587 | -0.036 | 0.046 | 0.424 |
| UBE2Q2 | chrUn30.8721302 | C | 838 | 0.040 | 0.044 | 0.369 | 586 | -0.014 | 0.053 | 0.793 |
| SHROOM3 | chrUn32.1032799 | C | 839 | 0.034 | 0.018 | 0.054 | 587 | 0.033 | 0.022 | 0.143 |

*SNP; single nucleotide polymorphism (Named from Illumina Feline Infinium Array); N: sample size; Beta: effect estimate from linear regression analysis; SE: standard error of Beta. (bold text indicates P-values < 0.05)*

**Table S5: KEGG pathways significantly enriched for renal trait Log Creatinine**

| **Term ID** | **Description** | **Count** | **Pathway size** | **P value** | **Genes** |
| --- | --- | --- | --- | --- | --- |
| hsa04024 | cAMP signaling pathway | 10 | 216 | 0.009178 | ADCY2/PDE3B/ATP2A2/MAPK1/GRIN2A/CREB5/CREB3L2/EP300/RAPGEF4/TIAM1 |
| hsa04928 | Parathyroid hormone synthesis, secretion and action | 7 | 106 | 0.009178 | ADCY2/MAPK1/ARHGEF1/PRKCB/MMP16/CREB5/CREB3L2 |
| hsa04935 | Growth hormone synthesis, secretion and action | 7 | 119 | 0.012676 | ADCY2/MAPK1/PRKCB/CREB5/CREB3L2/SOS2/EP300 |
| hsa04810 | Regulation of actin cytoskeleton | 9 | 218 | 0.016506 | RDX/ARHGEF12/MAPK1/LPAR1/ARHGEF1/PIP5K1C/SOS2/MYH9/TIAM1 |
| hsa05031 | Amphetamine addiction | 5 | 69 | 0.025979 | SLC18A2/PRKCB/GRIN2A/CREB5/CREB3L2 |
| hsa04261 | Adrenergic signaling in cardiomyocytes | 7 | 150 | 0.025979 | ADCY2/ATP2A2/MAPK1/CREB5/CREB3L2/PPP2R3C/RAPGEF4 |
| hsa04725 | Cholinergic synapse | 6 | 113 | 0.027791 | ADCY2/CHAT/MAPK1/PRKCB/CREB5/CREB3L2 |
| hsa04015 | Rap1 signaling pathway | 8 | 210 | 0.030427 | ADCY2/MAPK1/LPAR1/PRKCB/GRIN2A/PARD3/RAPGEF4/TIAM1 |
| hsa05030 | Cocaine addiction | 4 | 49 | 0.030427 | SLC18A2/GRIN2A/CREB5/CREB3L2 |
| hsa04911 | Insulin secretion | 5 | 86 | 0.030427 | ADCY2/PRKCB/CREB5/CREB3L2/RAPGEF4 |
| hsa05163 | Human cytomegalovirus infection | 8 | 225 | 0.030427 | ADCY2/ARHGEF12/MAPK1/ARHGEF1/PRKCB/CREB5/CREB3L2/SOS2 |
| hsa04540 | Gap junction | 5 | 88 | 0.030427 | ADCY2/MAPK1/LPAR1/PRKCB/SOS2 |
| hsa04728 | Dopaminergic synapse | 6 | 132 | 0.030427 | SLC18A2/PRKCB/GRIN2A/CREB5/CREB3L2/PPP2R3C |
| hsa04270 | Vascular smooth muscle contraction | 6 | 133 | 0.030427 | ADCY2/ARHGEF12/MAPK1/ARHGEF1/PRKCB/MYH9 |
| hsa05032 | Morphine addiction | 5 | 91 | 0.030427 | ADCY2/PDE3B/GRK3/PRKCB/GABRG1 |
| hsa05215 | Prostate cancer | 5 | 97 | 0.037736 | MAPK1/CREB5/CREB3L2/SOS2/EP300 |
| hsa04062 | Chemokine signaling pathway | 7 | 192 | 0.037866 | ADCY2/GRK3/MAPK1/PRKCB/SOS2/PARD3/TIAM1 |
| hsa04914 | Progesterone-mediated oocyte maturation | 5 | 100 | 0.037866 | CCNB1/ADCY2/PDE3B/MAPK1/MAD2L1 |
| hsa04916 | Melanogenesis | 5 | 101 | 0.037866 | ADCY2/MAPK1/PRKCB/CREB3L2/EP300 |
| hsa04072 | Phospholipase D signaling pathway | 6 | 148 | 0.038484 | ADCY2/MAPK1/LPAR1/PIP5K1C/SOS2/RAPGEF4 |
| hsa04922 | Glucagon signaling pathway | 5 | 107 | 0.041521 | ADCY2/PDE3B/CREB5/CREB3L2/EP300 |
| hsa05205 | Proteoglycans in cancer | 7 | 205 | 0.041521 | RDX/ARHGEF12/MAPK1/ARHGEF1/PRKCB/SOS2/TIAM1 |
| hsa04720 | Long-term potentiation | 4 | 67 | 0.041521 | MAPK1/PRKCB/GRIN2A/EP300 |
| hsa04218 | Cellular senescence | 6 | 156 | 0.041521 | CCNB1/BTRC/MAPK1/NBN/RBL1/TRPM7 |
| hsa05161 | Hepatitis B | 6 | 162 | 0.044727 | MAPK1/PRKCB/CREB5/CREB3L2/SOS2/EP300 |
| hsa04724 | Glutamatergic synapse | 5 | 114 | 0.044727 | ADCY2/GRK3/MAPK1/PRKCB/GRIN2A |
| hsa05165 | Human papillomavirus infection | 9 | 331 | 0.044727 | MAPK1/CREB5/CREB3L2/RBL1/SOS2/PPP2R3C/EP300/PARD3/UBR4 |
| hsa05166 | Human T-cell leukemia virus 1 infection | 7 | 219 | 0.047547 | ADCY2/MAPK1/RELB/CREB5/CREB3L2/MAD2L1/EP300 |
| hsa04022 | cGMP-PKG signaling pathway | 6 | 167 | 0.047744 | ADCY2/PDE3B/ATP2A2/MAPK1/CREB5/CREB3L2 |

*Term ID; KEGG pathway identification, Description; descriptor of enriched KEGG pathway, Count; number of feline genes enriched within given KEGG pathway, Pathway size; number of genes included in KEGG pathway, P value; significance, Genes; feline genes associated with enriched KEGG pathway*
